# Stage-dependent transcriptomic changes in human dermal fibroblast senescence model

**DOI:** 10.18632/aging.206371

**Published:** 2026-04-10

**Authors:** Michiko Kudo, Ryo Yonezawa, Hideaki Mizobata, Shigeharu Kinoshita, Shuichi Asakawa

**Affiliations:** 1 Laboratory of Aquatic Molecular Biology and Biotechnology, Department of Aquatic Bioscience, Graduate School of Agricultural and Life Science, The University of Tokyo, 1-1-1 Yayoi, Bunkyo-ku, Tokyo, 113-8657 Japan; 2 DHC Corporation Laboratories, Division 2, 2-42 Hamada, Mihama-ku, Chiba 261-0025, Japan

**Keywords:** cellular senescence, dermal fibroblasts, transcriptome analysis, immune–inflammatory signaling, aging biomarkers

## Abstract

Cellular senescence is a fundamental biological process that underlies organismal aging and age-related diseases; however, the temporal dynamics of gene expression during senescence remain unclear. This study investigated gene expression changes across aging progression and the molecular mechanisms involved using three replicative senescence models of human dermal fibroblasts, designated as young, middle, and old. RNA sequencing transcriptome analysis with two independent analytical pipelines revealed that the transcriptome profiles of the young and middle stages were similar, whereas that of the old stage was distinct. Differential expression analysis showed significant gene expression changes between the young and middle stages, with differentially expressed genes (DEGs) increasing as senescence progressed. STRING and Gene Ontology/Kyoto Encyclopedia of Genes and Genomes enrichment analyses indicated early activation of immune–inflammatory response clusters and decreases in structural maintenance genes. Using an alternative DEG set, non-negative matrix factorization (NMF) decomposed transcriptomes into distinct metagenes, with characteristic senescence-stage behaviors. NMF independently recapitulated immune–inflammatory activation and the loss of structural maintenance functions. These findings suggest that immune–inflammatory responses are engaged from early senescence, whereas cell adhesion and maintenance pathways decline progressively. These results identify early biomarkers and therapeutic targets, providing opportunities to delay or mitigate aging-related functional decline.

## INTRODUCTION

Aging, accompanied by several physiological changes, is the biological process during which the function of organisms declines as they mature [1–3]. Age-related decline in tissue and organ functions increases the risk of various diseases and substantially affects an individual’s lifespan [3]. Changes at the cellular level, particularly during cellular senescence, are likely involved in aging [4–6]. Cellular senescence is defined as a state of irreversible cell cycle arrest accompanied by distinct morphological and molecular changes [7]. Hallmark features include increased expression of cyclin-dependent kinase inhibitors, such as p16^INK4A^ and p21^Waf1/Cip1^, enhanced senescence-associated β-galactosidase (SA-β-gal) activity, and secretion of pro-inflammatory factors, collectively referred to as the senescence-associated secretory phenotype (SASP) [4]. Senescence can be categorized into replicative senescence (driven by telomere shortening after repeated cell divisions), stress-induced senescence (triggered by oxidative or genotoxic stress), and oncogene-induced senescence (resulting from aberrant mitogenic signaling) [8]. In experimental settings, additional senescence-inducing stimuli have been reported, such as specific drug treatments, oxidative stress, and DNA damage, which are frequently used to model stress-induced senescence *in vitro* [9–11]. Senescent cells accumulate in various tissues with age [12–15] and may adversely affect the surrounding tissue environment by secreting factors such as inflammatory cytokines (SASP), thereby promoting age-related diseases and organismal aging [16, 17]. Compared to normal young cells, senescent cells typically exhibit an enlarged and flattened morphology, altered plasma membrane composition, and nuclear structure changes, along with increased lysosomal content and mitochondrial alterations [8]. Cellular senescence contributes to aging at the tissue and organismal levels; however, it is a distinct biological process. Senescence refers to a cell-autonomous program characterized by permanent cell-cycle exit and specific molecular signatures. In contrast, aging denotes a multifactorial, systemic decline influenced by genetic, environmental, and lifestyle factors [8, 18]. Intrinsic aging, UV exposure-induced photoaging, and environmental stress drive the complex process of aging [19–22], and *in vitro* cell culture models are frequently used to analyze cellular responses and molecular mechanisms under specific conditions [23–25].

Aging is of considerable interest from health and cosmetic perspectives. Aging results in appearance changes [26–28], leading to high expectations for anti-aging products and interventions. Many ingredients and drugs are effective against aging, and some have been clinically applied [29–31]. These interventions can be broadly categorized into senolytics, which selectively induce apoptosis in senescent cells, and senomorphics, which suppress harmful senescence-associated phenotypes without eliminating the cells. Senolytics have shown promise in preclinical models by alleviating age-related tissue dysfunction and delaying the progression of various diseases. They may offer a transformative approach to treating age-associated conditions if proven safe and effective in clinical trials [17]. However, the mechanisms by which these ingredients and drugs exert their effects at the cellular level, particularly at the gene expression level, remain unclear. Experimental systems must accurately replicate aging to identify effective anti-aging ingredients and elucidate their mechanisms of action. Therefore, establishing model cells that accurately reflect age-related changes in human skin, enabling direct evaluation, is essential. A comprehensive understanding of the molecular changes associated with aging, achieved through a comparative analysis of gene expression profiles in senescent and non-senescent cells, is crucial for optimizing aging interventions and developing novel treatments. Although acute aging models induced by oxidative stress [32] or gamma irradiation [33] have traditionally been used, they do not adequately reflect the gradual changes associated with aging. These models often rely on strong, single stressors that trigger rapid and relatively uniform cellular responses, such as activation of DNA damage repair pathways or growth arrest. However, naturally senescent cells experience various chronic stresses—including DNA damage, protein misfolding, and mitotic errors—that accumulate gradually over time. These diverse and progressive signals lead to more complex and heterogeneous senescent phenotypes. Acute models may overrepresent stress-specific pathways and fail to capture the broader molecular and functional changes associated with replicative senescence and physiological aging [34, 35]. Therefore, this study adopted a replicative senescence model based on cumulative cell divisions, which better reflects the gradual nature of aging and is more suitable for investigating age-related gene expression dynamics and potential anti-aging interventions.

Population doubling level (PDL) was used as a proxy for cellular senescence to model gradual aging *in vitro*. PDL represents the cumulative number of cell divisions since initial plating and is widely used to track replicative senescence in cultured cells [7]. This study aimed to elucidate the gene expression changes associated with aging progression and the molecular mechanisms driving these dynamic changes by combining multiple methods. This study constructed a stepwise cellular aging model using human dermal fibroblasts undergoing replicative senescence, a cultured cell model that most effectively mimics *in vivo* aging and is physiologically the most realistic. This model was used to analyze the differences in gene expression profiles between senescent and non-senescent cells and to clarify their distinctive molecular basis.

## RESULTS

### Characteristics of cellular senescence *in vitro*

Three types of normal human dermal fibroblasts (NHDFs) with PDL obtained through subculture were used. These NHDFs were classified based on PDL as young (early passage, PDL ≤ 20), middle (middle passage, PDL 30–50), and old (late passage, PDL ≥ 60), according to the cumulative growth curve (Supplementary Figure 1) and culture duration and cumulative PDL values table (Supplementary Table 1). These ranges reflected consistent proliferation trends across the three senescence groups. To better reflect biological senescence status, classification was based on cumulative PDL rather than passage number, as passage number does not always reflect the actual replication history. For example, differences in seeding conditions may result in higher cumulative PDL despite fewer passages.

Cellular senescence was characterized by SA-β-gal staining, expression of senescence-associated protein markers (p21 [*CDKN1A*], p16 [*CDKN2A*], and p53 [*TP53*]), analyzed using western blotting, and SASP-related factors (interleukin [IL]-6 [*IL6*] and IL-8 [*CXCL8*]), analyzed via reverse transcription quantitative polymerase chain reaction (RT-qPCR). The SA-β-gal positivity rate increased with increasing PDL, and the proportion of SA-β-gal-positive cells was particularly high in the old group, which differed markedly from that in the young and middle groups (Figure 1A). p21, p16, and p53 protein expression increased with PDL. IL-6 and IL-8 mRNA expression also increased significantly, particularly in the old group (Figure 1B, 1C). These characteristics are consistent with those reported previously [13, 16, 36]; therefore, this *in vitro* model successfully reproduced the gradual cellular senescence that occurs in response to PDL.

**Figure 1:**
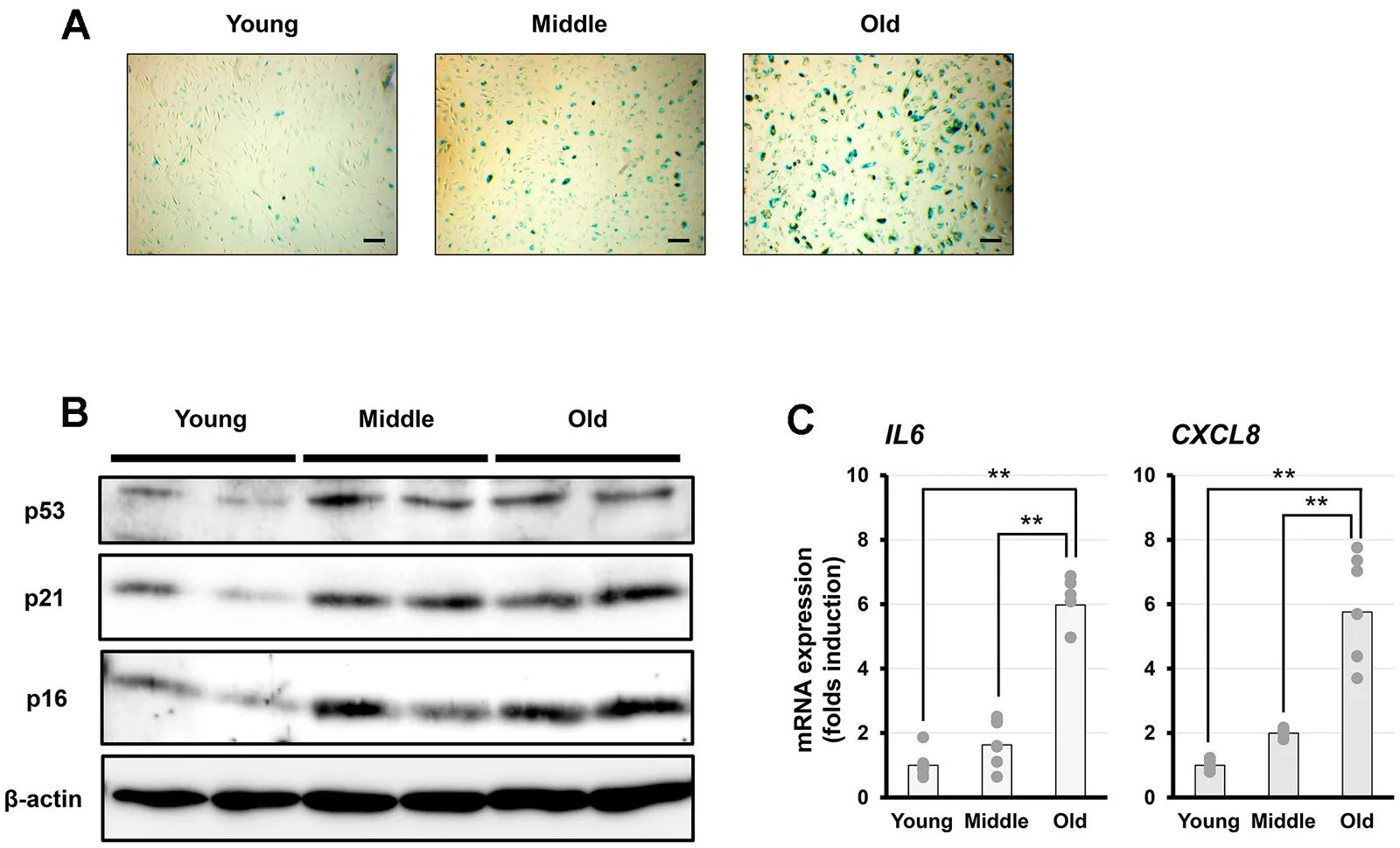
**Characterization of senescent cells *in vitro*.** (**A**) Representative images of senescence-associated β-galactosidase (SA-β-gal) staining in fibroblasts at different senescent stages (young, middle, and old). Scale bar = 200 μm. (**B**) Representative western blot analysis of p53, p21, and p16, with β-actin as a loading control. (**C**) PCR analysis of interleukin-6 (IL-6) and interleukin-8 (IL-8) expression. Data are presented as the mean ± standard error (SE) from seven independent experiments (n = 7). Statistical analysis was performed using the Tukey–Kramer test; **p < 0.01.

Segmented regression analyses were performed for eight senescence-related genes (*CDKN1A, CDKN2A, IL6, CXCL8, MMP1, MMP3, SERPINE1,* and *LMNB1*) to further validate the classification of NHDFs into young, middle, and old groups. Transcriptional breakpoints were identified for each gene along the PDL axis. Notably, six genes—*CDKN1A, IL6, CXCL8, MMP1, MMP3*, and *LMNB1*—exhibited breakpoints within the middle group, supporting its biological relevance as a transitional phase of early senescence. In contrast, *CDKN2A* and *SERPINE1* showed breakpoints within the old group, specifically at PDL ≈ 67–69, consistent with late-stage senescence. These results provide statistical support for interpreting the middle group as a biologically transitional phase consistent with early senescence. This transcriptional shift pattern reinforces the biological relevance of the middle group and complements the proliferation history and marker expression profiles described above (Supplementary Figure 2 and Supplementary Table 2).

### Transcriptome comparison by RNA sequencing

Total RNA was extracted from young, middle, and old senescent NHDFs (n = 7 per group), and RNA sequencing (RNA-seq) was performed to clarify the transcriptome changes associated with aging (Figure 2A). The DNBSEQ G-400 platform was used to obtain an average of approximately 75.6 million reads per sample, ensuring a sufficient sequencing depth for gene expression analysis.

**Figure 2:**
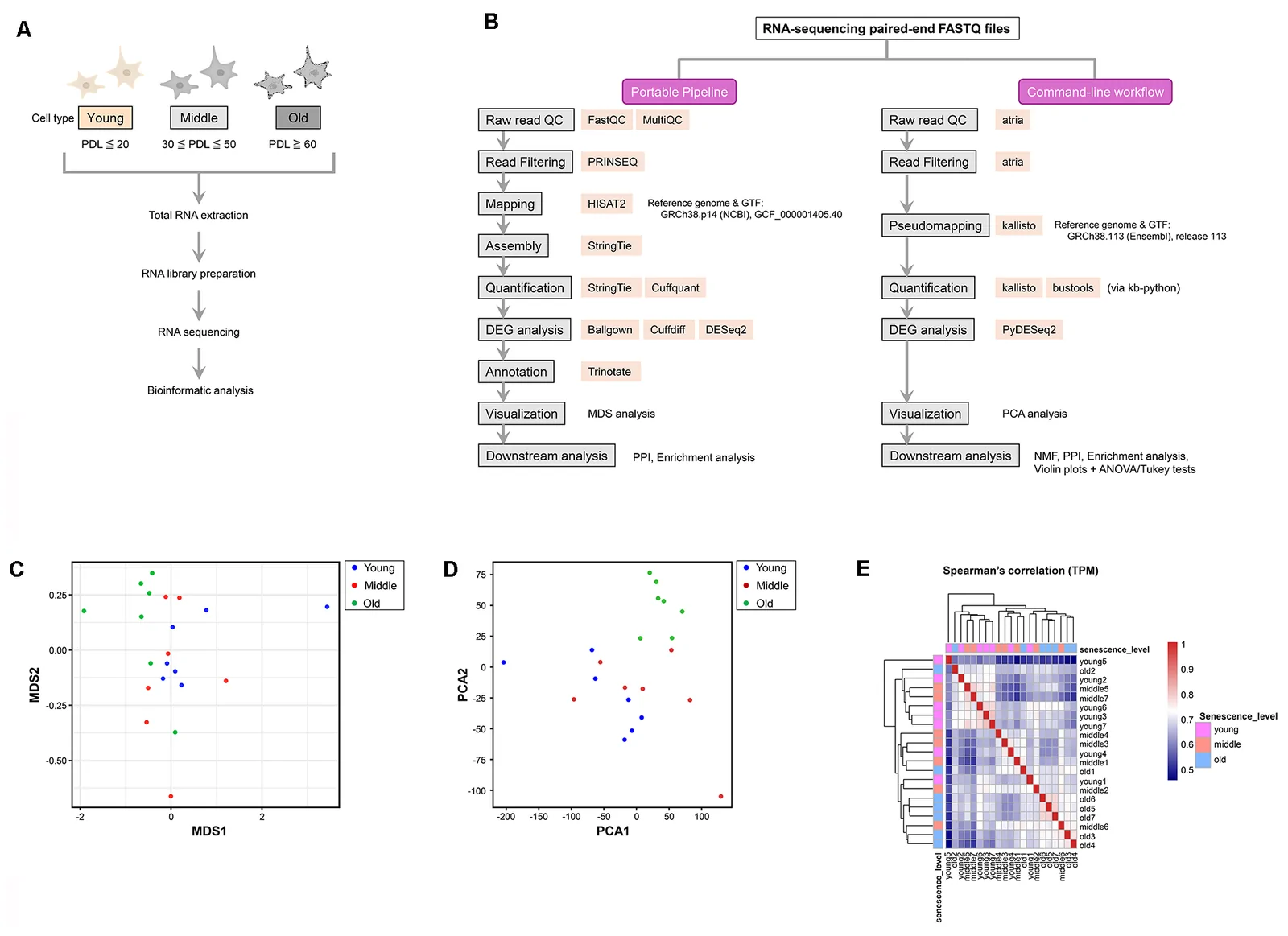
**Experimental workflow and visualization of transcriptomic similarity among samples.** (**A**) Experimental workflow for RNA sequencing (RNA-seq) analysis of young, middle, and old normal human dermal fibroblasts (NHDFs). (**B**) Comparative flowchart of the two RNA-seq analysis pipelines used in this study. The left panel illustrates the GUI-based Portable Pipeline, whereas the right panel shows the command-line workflow. Each step lists the tools used, highlighting methodological similarities and differences. (**C**) Multi-dimensional scaling (MDS) plot showing the pairwise dissimilarity of gene expression profiles across all samples. Each dot represents a single sample, and colors correspond to the aging stage: young (blue), middle (red), and old (green). (**D**) Principal component analysis (PCA) plot showing the similarity and dissimilarity of gene expression profiles across all samples. The horizontal (PC1) and vertical axes (PC2) represent the first and second principal components, respectively. Old samples are separated from the young and middle samples along the PC1 axis, whereas the young and middle samples overlap. (**E**) Spearman’s correlation heatmap of log_2_ (TPM + 1) values across all samples. Color intensity represents pairwise correlation coefficients, with sample IDs shown along both axes and labeled by senescence stage (young, middle, and old).

The resulting reads were then used for transcriptome quantification and comparative analysis using the automated analysis tool “Portable Pipeline,” and a general-purpose command-line workflow (Figure 2B). After multiple testing correction (adjusted p-value, padj), genes with significant intergroup differential expression changes were extracted using a threshold p of < 0.05. The results of the portable pipeline analysis are shown in Supplementary Table 3, and those of the PyDESeq2 analysis are shown in Supplementary Table 4. Supplementary Table 5 summarizes the differentially expressed genes (DEGs) commonly identified by both the portable pipeline and the PyDESeq2.

p16 (*CDKN2A*), p21 (*CDKN1A*), and Lamin B1 (*LMNB1*) were extracted from the DEG results of both analytical pipelines, and their expression profiles across senescence groups were compared to assess the behavior of canonical senescence markers. Differential expression analyses yielded inconsistent results: the portable pipeline detected *CDKN1A* only in the middle–old comparison, whereas the command-line workflow identified all three genes, albeit with partially discordant directions (Supplementary Table 6). As *CDKN2A*, *CDKN1A*, and *LMNB1* were not consistently detected as DEGs across pipelines, their transcripts per million (TPM) distributions were visualized using violin plots (Supplementary Figure 3). Violin plots revealed a significant difference only for *CDKN1A* between the middle and old groups, whereas *CDKN2A* and *LMNB1* showed non-significant trends. Because mean-based tests did not fully capture distributional differences, effect sizes were further quantified using Cliff’s delta. This analysis indicated non-monotonic trajectories for *CDKN2A* and *CDKN1A*, with decreased expression at the middle stage and increased expression at the old stage, whereas *LMNB1* showed a middle-stage increase followed by a decline in the old stage. Furthermore, the segmented regression analysis also included *CDKN2A* (Supplementary Figure 2B), *CDKN1A* (Supplementary Figure 2A), and *LMNB1* (Supplementary Figure 2H). Breakpoints for these genes were identified within the middle-to-old ranges, consistent with their phase-dependent trajectories observed in distributional and effect size analyses.

### Visualization of similarity using multi-dimensional scaling/principal component analysis

Multi-dimensional scaling (MDS) and principal component analysis (PCA) were performed to visualize the similarities and differences in gene expression profiles among the young, middle, and old groups. Both methods separated the old group from the young and middle groups (Figure 2C, 2D). The first principal component (PC1) and second principal component (PC2) explained approximately 35.4% and 14.4% of the total variance, respectively. The old group clustered distinctly along the PC1 axis. MDS plots visualizing pairwise differences among groups showed that the old group formed an independent cluster, consistent with the PCA results. These dimensionality reduction analyses were also used to visually assess sample-level variance before downstream analyses. No apparent outliers were observed, and within-group consistency was confirmed based on clustering patterns.

Spearman’s correlation analysis of log-transformed TPM values across all samples was performed to further confirm that the observed clustering was not driven by batch effects. Old samples clustered tightly together, consistent with the PCA/MDS results, whereas young and middle samples exhibited weaker within-group similarity and were not clearly separated from each other. Notably, no clustering by batch-associated metadata was observed (Figure 2E).

### Analysis of expression changes by comparing two groups

PCA and MDS plots showed that the old group was distinct from the young and middle groups, whereas the young and middle groups were not clearly separated (Figure 2C, 2D). However, this result does not necessarily indicate the absence of differences in gene expression between the two groups. Therefore, pairwise comparisons were conducted using a portable pipeline, in which gene expression levels between the young and middle groups, the middle and old groups, and the young and old groups were visualized with scatter plots. Significant DEGs were identified in all comparisons, and the number of these genes increased with cellular senescence (Figure 3A–3C and Supplementary Table 3). This finding suggests that senescence-related changes in gene expression profiles are characterized by large differences and by subtle changes in expression that gradually accumulate in the early stages of senescence.

**Figure 3:**
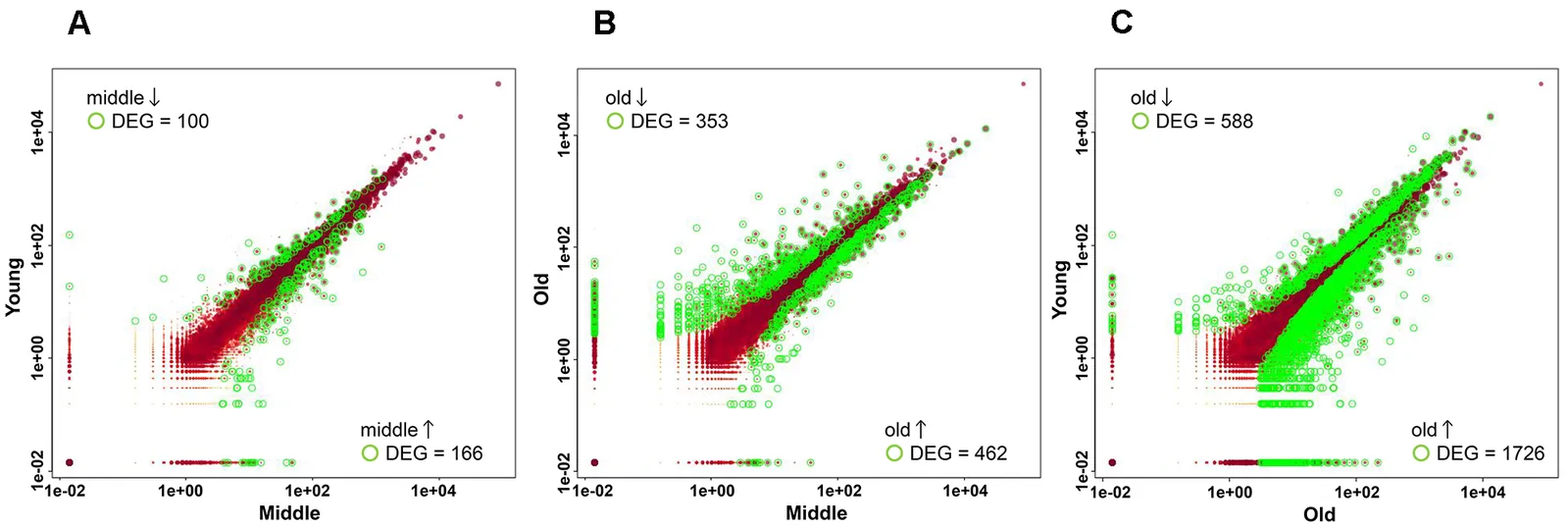
**Scatter plots showing differentially expressed genes (DEGs) in pairwise comparisons.** Scatter plots show the normalized expression levels of all genes in the two groups. Each dot represents a single gene. The expression levels of each gene were plotted on a log10 scale. Genes with significant differential expression (adjusted p-value < 0.05) are highlighted in green, whereas those with non-significant differential expression are shown in red. (**A**) Comparison between the young and middle groups. (**B**) Comparison between the middle and old groups. (**C**) Comparison between young and old groups.

### Protein–protein interaction network analysis and functional pathway identification using the STRING database

Protein–protein interaction (PPI) network analysis was performed using STRING to clarify the biological significance of the upregulated or downregulated genes in each comparison. PPI networks were constructed for the upregulated and downregulated genes in each comparison using the DEGs listed in Supplementary Table 3, and multiple functionally related clusters were identified (Figure 4). The upregulated genes in the young-to-middle, middle-to-old, and young-to-old comparisons formed one, three, and five clusters, respectively. The downregulated genes formed three, three, and five clusters, respectively. Functional annotation of each cluster was performed using Reactome pathways, and the top five Reactome pathway terms (term IDs) for each cluster are summarized in Supplementary Table 7.

**Figure 4:**
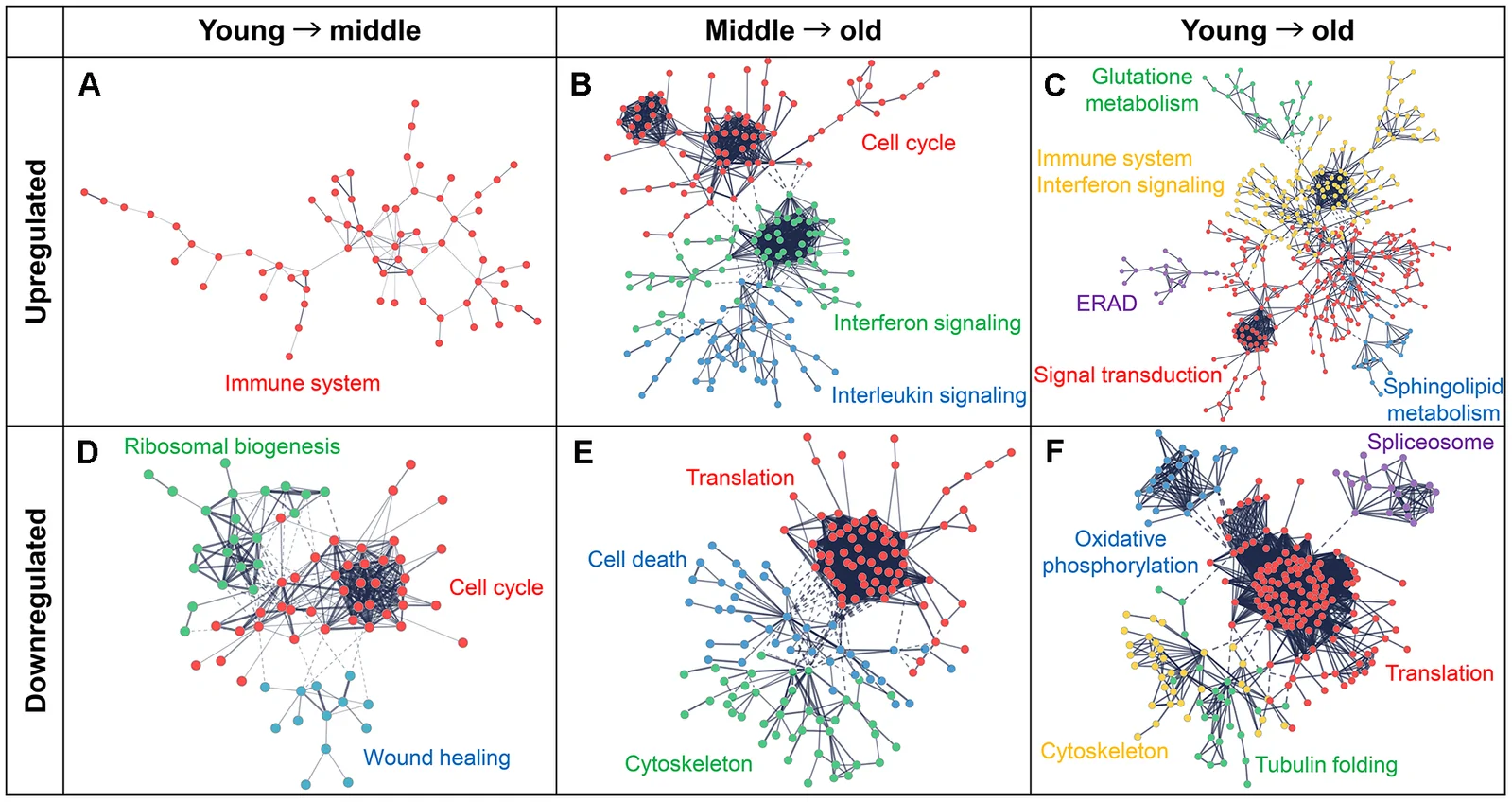
**Protein–protein interaction (PPI) networks and functional clusters of DEGs.** PPI networks were constructed using the STRING database. Nodes represent genes and edges represent their interactions. Upregulated genes are shown in the upper panels (**A**–**C**) and downregulated genes are shown in the lower panels (**D**–**F**). Functionally enriched clusters are highlighted in different colors and labeled with the corresponding pathway or process names. (**A**, **D**) Networks for the comparison between the young and middle groups. (**B**, **E**) Networks for comparison between middle to old groups. (**C**, **F**) Networks used for comparison between young and old groups.

Cluster labels in Figure 4 were assigned based on the predominant functional category represented by these pathways. The upregulated genes in the young-to-middle comparison formed a single cluster primarily related to the immune system and inflammatory response (Figure 4A). These functions were consistently enhanced in the middle-to-old and young-to-old comparisons (Figure 4B, 4C). Specific pathways, such as interferon and IL signaling, were identified in the comparison between the middle and old groups. In contrast, the downregulated genes were predominantly involved in homeostasis, including cell cycle, translation, wound healing, cell adhesion, and cytoskeleton, and were widely decreased with the progression of aging (Figure 4D–4F).

### Gene ontology and Kyoto encyclopedia of genes and genomes pathway enrichment analysis

Biological process Gene Ontology (GO) and Kyoto Encyclopedia of Genes and Genomes (KEGG) pathway analyses were performed for each cluster using Enrichr software (Figure 5). The results for the clusters classified as the immune and inflammatory response systems in Figure 4 are shown in Figure 5. The results for all clusters, including the immune-inflammatory clusters, are provided in Supplementary Figure 4A–4F. The immune-related cluster from young-to-middle was significantly enriched for innate immune response in GO and for viral infection and cytokine-related pathways in KEGG (Figure 5A). In the middle-to-old cluster, interferon-related pathways were highly enriched (Figure 5B). In contrast, in the young-to-old cluster, antiviral responses and pathogen recognition pathways such as nucleotide-binding domain (NOD)-like receptor were highly enriched (Figure 5C). These Enrichr results are consistent with the network analysis using STRING and support the hypothesis that the activation of the immune and inflammatory systems progresses from the early stages of senescence.

**Figure 5:**
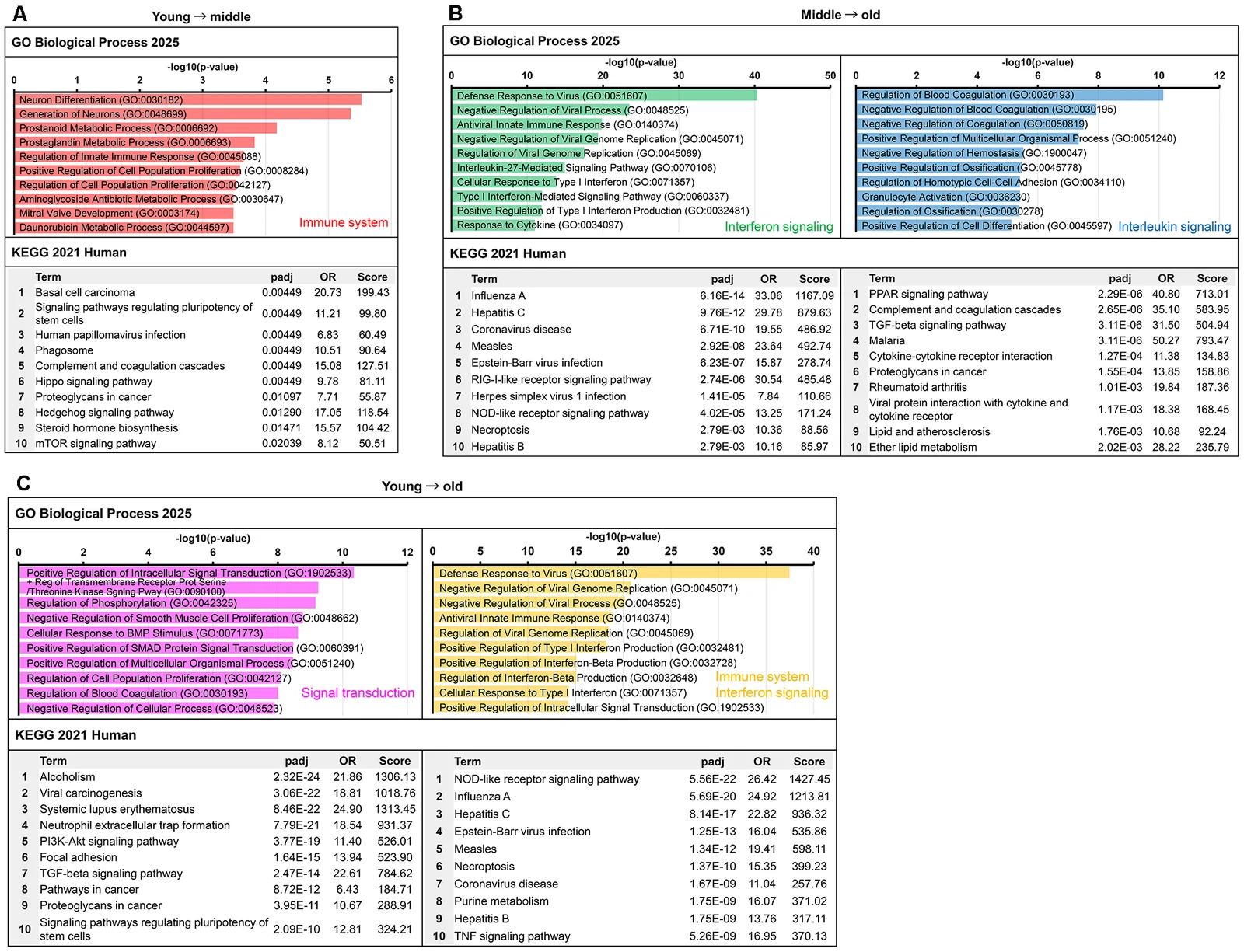
**Functional enrichment analysis of immune–inflammation-related clusters identified from senescence progression.** Enrichment results from Gene Ontology (GO) biological process and Kyoto Encyclopedia of Genes and Genomes (KEGG) pathway analyses for clusters classified as immune- or inflammation-related in the comparisons between the young and old groups. Bar colors correspond to the cluster colors in Figure 4A–4C: red, immune system (Figure 4A); green, interferon signaling (Figure 4B); blue, IL signaling (Figure 4B); pink, signal transduction (Figure 4C); and orange, immune system/interferon signaling (Figure 4C). The top 10 terms ranked by adjusted p-values are shown for each cluster. (**A**) Clusters upregulated from the young to middle. (**B**) Upregulated clusters from middle to old. (**C**) Clusters upregulated from young to old.

### Non-negative matrix factorization analysis

To examine the heterogeneity within a dataset, non-negative matrix factorization (NMF) analysis is used to decompose gene expression data into a small number of independent expression programs (metagenes). NMF analysis was performed to decompose the expression data into six metagenes (Meta1–6) (Figure 6A) using the DEG results (Supplementary Table 4). The optimal number of metagenes (K) was determined using evaluated cophenetic correlation, consistency metrics, and iteration stability across a range of K values. In addition to these statistical criteria, we compared how clearly each K value separated stage-dependent expression patterns across the young, middle, and old groups. Although K=5 showed relatively high cophenetic and consensus values, it did not clearly distinguish stage-specific programs. In contrast, K=6 yielded well-separated metagenes corresponding to young-, middle-, and old-enriched expression patterns, providing the best balance between statistical stability and biological interpretability (Supplementary Figure 5A–5D). In the heatmap, the contribution of Meta2 increases in the order young → middle → old, whereas Meta4 decreases, and Meta6 is highest in the middle group (Figure 6A). Meta1 contributed significantly to all the samples and was not associated with senescence-related changes (Figure 6A and Supplementary Figure 6). The top-ranked genes from each metagene for STRING and Enrichr analyses were extracted for functional annotation. Because the structural characteristics of metagenes differed, the optimal number of top genes varied (Meta2: 100, Meta4: 200, and Meta6: 500). Meta2 exhibited a well-defined structure that was fully captured by the top 100 genes. Meta4 contained an overrepresentation of rRNA-related genes among the highest-ranking features, necessitating a broader set to recover an interpretable cluster structure. Meta6 encompassed diverse extracellular matrix (ECM)- and adhesion-related programs, for which more genes (n = 500) were required to adequately represent the heterogeneity of functional annotations. These threshold adjustments ensured that functional interpretations remained robust and biologically meaningful across metagenes (Figure 6B, 6C). STRING functional annotation was based on UniProt Annotated Keywords.

**Figure 6:**
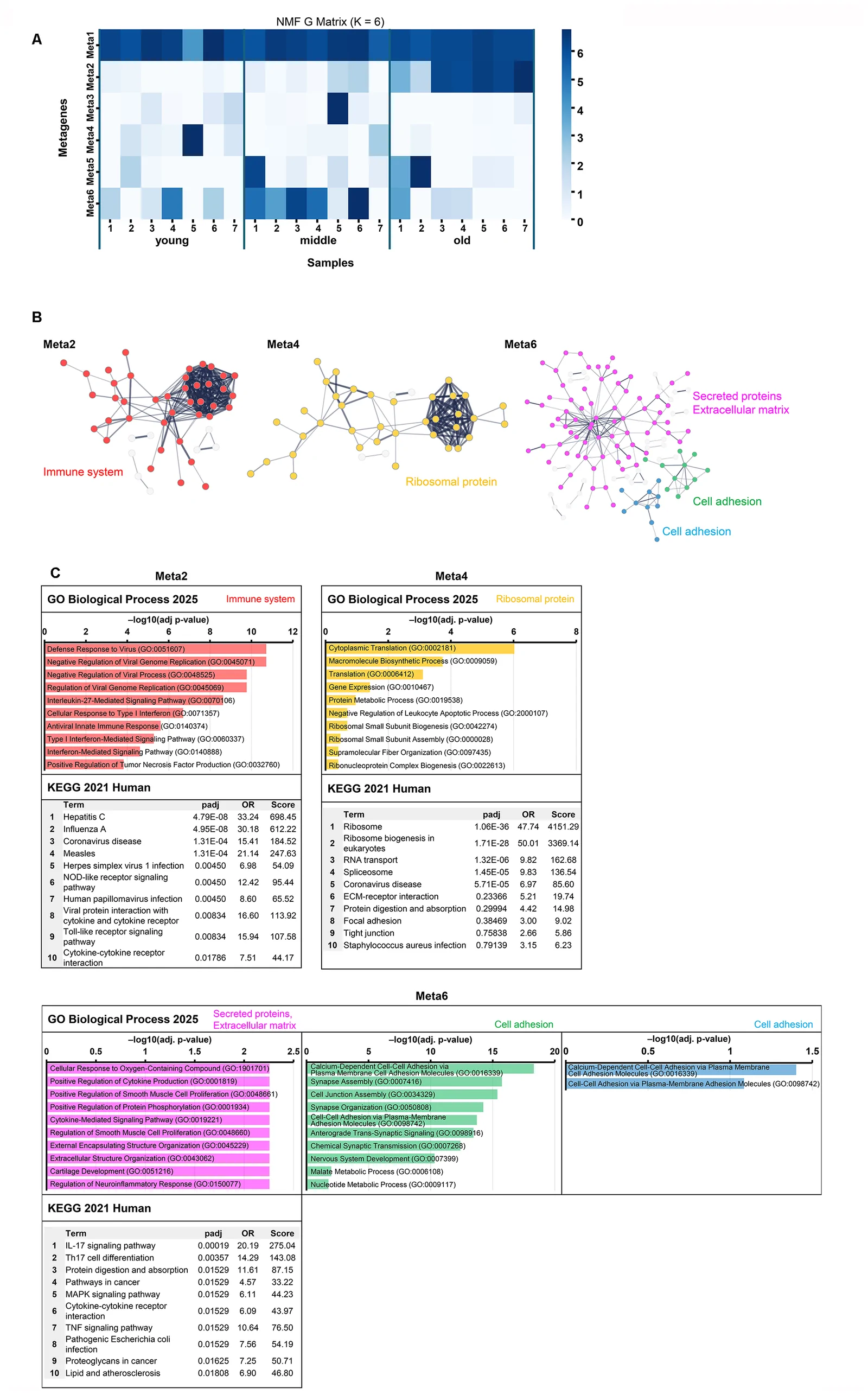
**Non-negative matrix factorization analysis of gene expression data and functional characterization of metagenes.** (**A**) Heatmap of metagene expression patterns across samples. This shows the contribution of each metagene (Meta1–6) to the gene expression profile of each sample (young, middle, and old). The contribution of Meta2 increased with senescence, that of Meta4 decreased, and that of Meta6 peaked in the middle group. (**B**) STRING network. PPI networks were constructed for the top 100 genes of Meta2, the top 200 genes in Meta4, and the top 500 genes of Meta6. Nodes represent proteins (Meta2: red, Meta4: orange, Meta6: pink/green/blue), and edges represent PPI. Functionally enriched clusters are highlighted and annotated based on UniProt keywords. Nodes outside the annotated functional clusters (based on UniProt keywords) were retained in the network but shown in gray to visually distinguish them from the main modules. (**C**) Functional enrichment analysis of Meta2, Meta4, and Meta6 using GO Biological Process (top) and KEGG Human (bottom). GO enrichment results are shown as horizontal bar graphs, with bar length representing –log10 (adjusted p-value). Bar colors correspond to the cluster colors in panel B. KEGG tables list the top 10 terms ranked by adjusted p-value. Other metagene enrichment results are shown in Supplementary Figure 7.

Meta2 formed a single cluster related to the immune system. GO/KEGG analysis also showed enrichment for the antiviral response, interferon, Toll-like receptor/NOD, and cytokine pathways (Figure 6B, 6C). Meta4 was dominated by rRNA genes and was enriched in translation and ribosome-related pathways. This reduced contribution suggested decreased translational activity (Figure 6C). Meta6 formed three clusters of the top 500 genes. Cluster 1 was dominated by ECM/connective tissue remodeling and cytokines, such as IL-17/tumor necrosis factor-related pathways. Both clusters 2 and 3 were dominated by cell adhesion-related GO keywords (Figure 6B, 6C).

Based on NMF analysis, these findings were consistent with the portable pipeline-based results (Figures 4, 5). The results for the remaining metagenes (Meta1, 3, and 5), which did not show clear senescence-related associations, are provided in Supplementary Figure 7. These results demonstrate that the activation of immune and inflammatory responses and decreased translation-related functions associated with cellular senescence were consistently detected across different analytical and gene extraction methods.

### Senescence-stage–dependent expression profiles of representative genes

Nine representative genes were selected based on their functional relevance and contribution to metagenes to further characterize senescence-associated transcriptomic changes. Genes associated with immune/inflammatory responses (Meta2: *IFIT1*, *MX1*, and *OAS1*), translational regulation (Meta4: *EIF3CL*, *EEF1D*, and *RPLP2*), and ECM/adhesion (Meta6: *MMP9*, *SULF1*, and *PCDHGB2*) were prioritized.

Expression distributions across the young, middle, and old groups were visualized using violin plots with individual data points overlaid (Figure 7). Significance was assessed via one-way analysis of variance (ANOVA) followed by Tukey’s post-hoc test. Significant senescence-stage-dependent differences were observed in *IFIT1*, *MX1*, and *OAS1*, which were upregulated in the old group compared to the young and middle groups. In contrast, *EIF3CL*, *EEF1D*, *RPLP2*, *MMP9*, *SULF1*, and *PCDHGB2* did not reach significance. Nevertheless, effect size analyses (Cohen’s d) and detection rates revealed non-significant but measurable shifts in several of these genes (Supplementary Table 8). These results indicate that although statistical significance was limited to Meta2 genes, additional analyses provided complementary evidence of senescence-associated changes in Meta4 and Meta6 genes.

**Figure 7:**
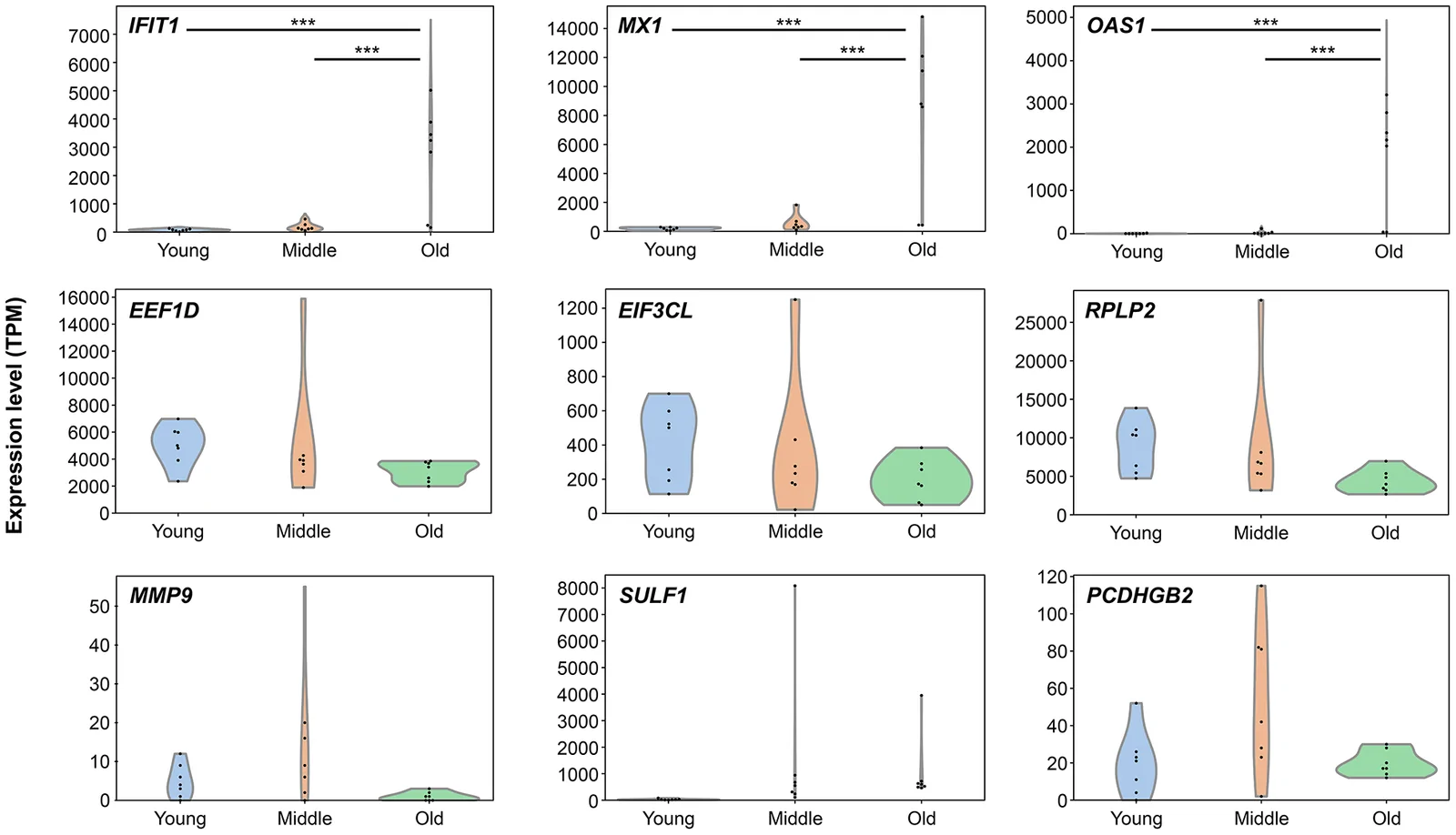
**Senescence-stage-dependent expression patterns of representative genes.** Violin plots showing transcript expression levels (TPM) of nine selected genes across three senescence stages: young, middle, and old. Genes include *IFIT1*, *MX1*, *OAS1* (Meta2), *EIF3CL*, *EEF1D*, *RPLP2* (Meta4), and *MMP9*, *SULF1*, *PCDHGB2* (Meta6). Each plot displays the distribution and individual data points. Statistical significance was assessed using one-way analysis of variance followed by Tukey’s post-hoc test. Asterisks indicate significant differences between groups: *p < 0.05, **p < 0.01, ***p < 0.001.

## DISCUSSION

In this study, MDS and PCA analysis confirmed global differences in the expression patterns among the young, middle, and old groups. A portable pipeline and general-purpose command-line tools were used to ensure analytical robustness, focusing on identifying common findings. Significant changes were observed in the gene expression profiles, particularly in the old group, indicating that the progression of replicative senescence induces large-scale changes throughout the transcriptome. In contrast, the gene expression profiles of the young and middle groups were relatively similar. This result is consistent with the lack of significant differences in IL-6 and IL-8 levels between the young and middle groups, supporting the validity of the analysis results. Sample-level variance was visually assessed using PCA and MDS plots before downstream analyses. Consistent with these dimensionality reduction analyses, Spearman’s correlation analysis also showed that old samples clustered tightly, whereas young and middle samples were not clearly separated. No clustering by batch-associated metadata was observed, and no apparent outliers were detected, with within-group consistency confirmed based on clustering patterns. However, because the middle group represents a transitional stage, it may exhibit biological heterogeneity not fully captured by dimensionality reduction alone. Future studies should incorporate additional replicates and formal variance metrics to validate group-level consistency.

Although the MDS/PCA did not separate the young and middle groups, this result did not necessarily mean that no molecular-level differences existed between the two groups. Therefore, DEG analysis based on a two-group comparison was performed, and numerous significantly altered genes were identified, including when comparing the young and middle groups. Segmented regression models were applied to eight senescence-related genes to estimate transcriptional breakpoints along the PDL axis. Six genes exhibited breakpoints within the middle group, while two genes showed breakpoints in the old group. Bootstrap analyses indicated that the 95% confidence intervals of most genes overlapped with the middle range, whereas *CDKN2A* and *SERPINE1* extended into the old range. These results provide statistical support for interpreting the middle group as a biologically transitional stage consistent with early senescence, suggesting that molecular changes associated with early-stage cellular senescence are already detectable in this group. Simultaneously, the distributional shifts highlight marker-specific timing differences, with some canonical signatures consolidating later in the old group. This heterogeneity is consistent with a phased model of senescence—early immune–inflammatory activation emerging in the middle stage, with certain cell-cycle and ECM-associated changes stabilizing later in the old group. However, canonical senescence markers (*CDKN2A*, *CDKN1A*, and *LMNB1*) did not exhibit fully consistent or monotonic changes across pipelines, and distributional analyses revealed non-linear trajectories. These non-monotonic patterns reflect the phased nature of senescence. Early senescence (middle stage) is a highly dynamic transition period in which stress-response, inflammatory, and ECM-remodeling pathways peak before stabilizing in late senescence. Fully senescent cells (old stage) exhibit reduced transcriptional plasticity, which can result in expression levels that appear similar to those in young cells despite representing a fundamentally different biological state. Thus, genes showing a middle-stage maximum or minimum do not indicate a reversal of senescence in the old stage, but rather reflect the transient activation or suppression of pathways that are most dynamic during the transition into senescence and become stabilized once full senescence is established. This raised the question of how to interpret their behavior in the context of senescence progression. Notably, these genes were also included in the segmented regression analysis, which identified breakpoints within the middle-to-old ranges. Collectively, these results support the view that the middle group represents a phase of dynamic regulation rather than a simple intermediate state. Thus, consistent with this interpretation, the middle group may represent an early stage of cellular senescence in which some molecular changes are still potentially reversible. This hypothesis is supported by previous studies showing that partial reprogramming can restore cellular plasticity in aged tissues [37]. Additionally, Kim et al. [38] demonstrated that SLIT2 derived from young fibroblasts can attenuate senescent features in mid-old fibroblasts, further suggesting that cells at this stage retain some plasticity. These findings provide external support for the interpretation that the middle group represents an intermediate, modifiable state. In contrast, the old group exhibits more stable and likely irreversible features of senescence, indicating that the aging process has progressed beyond the stage at which molecular changes can be easily modified.

Genes related to immune responses and inflammatory signaling were significantly upregulated in the young vs. middle group, suggesting that the immune–inflammatory system may be activated early in aging. This trend was more pronounced in comparisons between the middle and old groups and between the young and old groups, where aging was more advanced. In contrast, pathways essential for maintaining basic cellular homeostasis, such as ribosomal biogenesis, translation, oxidative phosphorylation, and spliceosomes, were downregulated with the progression of aging. These results highlight the dual nature of aging, characterized by a gradual increase in immune and inflammatory responses, alongside a gradual decline in cellular function. This result was further supported by the extraction of transcriptional programs using NMF analysis.

Several metagenes exhibited characteristic behavior as aging progressed. Meta2 was enriched in immune response-related genes and showed a stepwise increase in contribution from young to middle to old age. This finding is consistent with the activation of inflammation-related genes observed in the RNA-seq analysis and may reflect the senescence-dependent enhancement of the immune–inflammatory system, consistent with the RNA-seq and RT-qPCR results. Meta4, comprising translation and ribosomal genes, showed a decreased contribution with age, supporting a decline in protein synthesis and basic cellular functions associated with aging. Meta6 was enriched in cell adhesion- and ECM-related genes, with the highest contribution observed in the middle group, likely reflecting substantial changes in the extracellular environment and tissue remodeling occurring in the early stages of senescence. Therefore, each NMF metagene reproduces the multilayered nature of senescence, including the activation of the immune–inflammatory system, decline in basic cellular functions, and early-senescence-specific remodeling.

Representative gene trajectories highlight the multilayered nature of senescence progression. Immune and inflammatory response genes (Meta2: *IFIT1, MX1*, and *OAS1*) exhibited a stepwise increase from young to middle to old, establishing immune activation as a defining hallmark of early senescence. This trajectory suggests that immune–inflammatory pathways are not restricted to late stages but emerge during the transitional middle phase, positioning them as central drivers of senescence initiation. Translational regulation genes (Meta4: *EIF3CL, EEF1D,* and *RPLP2*) showed non-significant downward tendencies. Their variability in the middle stage suggests that translational repression accompanies, but does not dominate, the onset of senescence, reflecting the instability of early-stage cellular states. ECM- and adhesion-related genes (Meta6: *MMP9, SULF1,* and *PCDHGB2*) peaked in the middle stage, consistent with the NMF analysis results. Although statistical tests did not confirm significance, this transient remodeling complements immune activation, indicating that structural reorganization contributes to the transitional features of early senescence.

Collectively, these findings reveal a layered choreography of senescence: immune activation emerging as the central hallmark of early stages, translational decline and extracellular remodeling acting as supporting processes, and all converging to shape the senescence-stage–dependent transcriptomic landscape. Notably, these *in vitro* trajectories provide a conceptual bridge to organismal aging, offering mechanistic insight into how immune priming, functional decline, and structural remodeling collectively drive tissue deterioration and inflammaging.

In contrast, Meta1, comprising ECM- and ribosome-related genes, showed a high contribution across all samples. However, no senescence-dependent behavior was observed. Therefore, Meta1 is unlikely to regulate the progression of aging directly, but rather represents a universal expression network underlying cellular homeostasis. Meta3 and Meta5 were not investigated in this study as they did not show a clear aging dependence.

The activation of immune and inflammatory responses and the decline in translation function associated with aging in human dermal fibroblasts were consistently supported by multiple analytical methods. Fibroblast senescence can promote inflammatory aging in local tissues and throughout the body via the SASP [39]. Consistently, in this study, typical SASP-related genes, such as *IL6* and *CXCL8*, were markedly upregulated in old fibroblasts compared with young ones. In contrast, middle fibroblasts showed an intermediate pattern without significant differences from the young group. This trend is consistent with the MDS/PCA results, supporting the notion that fibroblasts enter an early stage of senescence in the middle group before progressing to full senescence in the old group. As fibroblast senescence contributes to local skin inflammation and aging-related inflammation, fibroblasts are essential target cells for aging control, further highlighting the importance of using human dermal fibroblasts as targets for the stepwise analysis in this study. Additionally, the elevated interferon- and cytokine-related pathways in the middle group suggest that fibroblasts acquire SASP-like features earlier than the stage at which classical senescence markers become prominent. SASP develops in a stepwise manner [8, 40]; early phases are dominated by interferon-stimulated genes and chemokine signaling, whereas later phases include the full secretory phenotype [41, 42]. Consistent with previous reports, the transcriptional activation observed in the middle group is consistent with an emerging “early SASP” state or priming phase, reflecting a progressive rather than binary transition. This concept also aligns with the model of inflammaging, in which low-grade chronic inflammation progressively intensifies over time [43, 44]. These findings provide a transcriptomic basis for the contribution of dermal fibroblasts to inflammaging during replicative aging.

Previous acute aging models, such as those using oxidative stress or radiation exposure [32, 33], have primarily focused on the dichotomy between young and old, making it difficult to detect subtle changes in the intermediate stages. In contrast, tracking replicative senescence over time reveals that, at the molecular level, inflammation-related pathways are already activated in the early stages of aging and become irreversibly fixed in later stages. Kim et al. [38] compared young, mid-old, and old cells and demonstrated the upregulation of pro-inflammatory genes and downregulation of anti-inflammatory genes, based on multifaceted validation including *in vivo* studies. However, the mid-old group included a relatively advanced state of aging, which emphasized the dichotomy between young and old. In contrast, in the present study, the middle group was positioned closer to the younger stage, allowing for the detection of subtle changes during the early stages of aging. Furthermore, aging-associated expression changes were extracted as metagenes, and the gradual activation of the immune–inflammatory system and the decline in translational function were systematically analyzed. These results elucidate the complex interactions driving aging, which traditional pathway enrichment analysis could not fully capture, thereby highlighting that a single factor does not cause aging but rather represents a hierarchical and multilayered system.

This study had some limitations that must be acknowledged. First, the validation efforts were restricted to mRNA-level confirmation. Although IL-6 and IL-8 were validated at the transcript level using RT-qPCR, protein-level confirmation for other immune- or structural-related genes was not conducted. As mRNA–protein correlation varies substantially depending on gene function and cellular context, incorporating enzyme-linked immunosorbent assay, western blotting, or proteomic approaches in future studies is essential for strengthening the functional interpretation of the transcriptomic signatures identified in this study. Second, although the findings suggest that immune–inflammatory system activation may play a central role in aging, key genes and signals remain unidentified. Third, the study was limited to the transcriptome and did not include multiple factors involved in aging control, such as miRNAs, long non-coding RNAs, pseudogenes, transposons, and exosomes. These factors interact with each other and form networks that regulate gene expression and cellular functions [45–50]. Building on these findings, it can be further hypothesized that miRNAs regulate their target mRNAs, while lncRNAs and pseudogenes may influence these interactions, potentially forming complex feedback loops that modulate aging-related processes. Fourth, although classical senescence markers such as SA-β-Gal, p16, and p21 were evaluated, proliferation markers (e.g., Ki67) were not assessed at the protein level. However, transcriptomic analysis revealed significant changes in *MKI67* expression: a decrease from young to middle, a transient increase from middle to old, and an overall decrease from young to old. The transient increase observed between middle and old may reflect heterogeneity within the middle group or stress-related proliferative responses. While these transcriptomic results support reduced proliferation overall during replicative senescence, protein-level validation is lacking. Future studies will include proliferation markers such as Ki67 by immunostaining to further distinguish replicative senescence from density-dependent quiescence. Fifth, all experiments were conducted using primary human dermal fibroblasts derived from a single neonatal donor. This approach was selected to minimize inter-individual variability and to ensure consistent tracking of replicative senescence across defined PDL stages. However, this design limits the generalizability of the findings, as it does not account for donor- or cell-type-specific differences in senescence trajectories. Future studies incorporating fibroblasts from multiple donors and additional cell types are essential to validate the universality of the stage-specific transcriptomic signatures identified in this study.

In conclusion, this study demonstrates that the activation of the immune–inflammatory system is a central hallmark of early senescence in human dermal fibroblasts, while translational decline and extracellular remodeling act as complementary processes shaping the multilayered nature of senescence progression. These findings, derived from transcriptomic analyses and representative gene validation, suggest that immune–inflammatory activity may be associated with cellular senescence. The metagenes identified in this study provide valuable insights into transcriptomic shifts during senescence progression and may inform future research on cellular senescence. Moreover, controlling aging-related molecular pathways may play a role in the development of pharmaceuticals and biomarkers, as well as in the design of familiar anti-aging products, such as cosmetics and health foods. However, such applications require further investigation. As skin fibroblasts actively shape the local immune–inflammatory environment and may even influence systemic signaling, pathways associated with fibroblast-driven inflammatory regulation could represent promising avenues for future aging-related interventions. This study primarily investigated and discussed transcriptomic changes at the mRNA level. However, aging-associated transcriptional signatures may also be driven by upstream mechanisms such as epigenetic modifications, metabolic reprogramming, and secretory activity. These processes can shape chromatin accessibility, energy balance, and paracrine signaling, thereby influencing the observed mRNA patterns. Therefore, future studies should integrate multilayered omics approaches—including epigenomic, metabolomic, and proteomic profiling—together with *in vivo* validation, to fully elucidate the complex regulatory networks underlying aging progression. Nevertheless, although these possibilities remain speculative, the notion that immune–inflammatory pathways represent a major driver of early senescence is supported by the findings of this study. This insight provides an important conceptual basis for understanding the multilayered nature of aging and may serve as a starting point for evaluating the effectiveness of targeting immune–inflammatory processes in aging research and intervention strategies.

## MATERIALS AND METHODS

### Cell culture

NHDFs were isolated from the neonatal foreskin and purchased from Thermo Fisher Scientific (Waltham, MA, USA). The cells were cultured in FI200 medium (Gmep Inc., Fukuoka, Japan) supplemented with l-glutamine (Thermo Fisher Scientific) at 37° C in a humidified incubator with 5% CO_2_. All experiments were performed using NHDFs derived from a single donor line, and biological replicates were generated from independent passages of this line. Depending on the number of cell divisions, the senescence levels of the cells were defined as young (PDL ≤ 20), middle (30 ≤ PDL ≤ 50), and old (PDL ≥ 60). To minimize contact inhibition, subculturing was consistently performed at 70–80% confluency throughout passaging, ensuring comparable growth conditions across all stages. Cumulative PDL values and culture duration were calculated from cell counts across passages, and used to plot the growth curve (Supplementary Figure 1 and Supplementary Table 1).

### SA-β-gal staining

SA-β-gal staining was performed using a Senescence Detection Kit (Abcam, Cambridge, UK) according to the manufacturer’s instructions. The Staining Solution Mix was incubated at 37° C for 1 h to overnight. However, the cells were incubated for up to 3 d in this study to enhance blue color development. The cells were observed using a DM IL HC IL-2 microscope (Leica Microsystems, Wetzlar, Germany), and blue staining was confirmed.

### Western blotting analysis

Whole cell lysates were prepared on ice using 1× RIPA buffer (50 mM Tris-HCl, pH 7.5, 150 mM NaCl, 1% NP-40, 0.5% sodium deoxycholate, 0.1% SDS, 1 mM EDTA, and 10 mM NaF) supplemented with cOmplete™ Tablets, Mini EDTA-free, and Protease Inhibitor Cocktail (Merck KGaA, Darmstadt, Germany). Cell lysates were centrifuged at 20,000 × *g* (approximately 15,000 rpm) for 10 min at 4° C to pellet the insoluble material. The supernatant was collected as the total cell lysate. After centrifugation, the protein concentrations of the supernatants were determined using Pierce™ BCA Protein Assay Kits (Thermo Fisher Scientific). All samples were prepared to a uniform concentration. The sample buffer (250 mM Tris-HCl, pH 6.8, 20% mercaptoethanol, 8% SDS, 20% sucrose, and 0.1% bromophenol blue) was used for western blotting. The lysates were separated using sodium dodecyl sulfate-polyacrylamide gel electrophoresis and transferred onto polyvinylidene fluoride membranes (Merck KGaA). Membranes were incubated with blocking buffer at room temperature for 1 h. The primary antibodies were incubated overnight at 4° C, followed by incubation with appropriate horseradish peroxidase (HRP)-conjugated secondary antibodies. Blots were developed with ImmunoStar LD (FUJIFILM Wako Pure Chemical Corporation, Osaka, Japan). Signals were detected using an ImageQuant™ LAS 4000 Biomolecular Imager (Cytiva, Marlborough, MA, USA). The primary and secondary antibodies used were as follows: anti-p53 (#2524, Cell Signaling Technology, Danvers, MA, USA), anti-p21^Waf1/Cip1^ (#2947), anti-p16^INK4A^ (#92803), anti-beta-actin (ab8227, Abcam), HRP-linked anti-rabbit IgG (#7074, Cell Signaling Technology), and HRP-linked anti-mouse IgG (NA931, Cytiva).

### RT-qPCR analysis

Total RNA isolation and cDNA synthesis were performed using a RNeasy Mini Kit (74106, QIAGEN, Venlo, Netherlands) and PrimeScript™ II 1st strand cDNA Synthesis Kit (6210A, TAKARA BIO, Shiga, Japan), respectively. Total RNA was reverse transcribed using random hexamers and the reverse transcriptase included in the kit. RT-qPCR was performed using PrimeTime™ Gene Expression Master Mix and PrimeTime^TM^ Predesigned qPCR Assays (Integrated DNA Technologies, Inc., Coralville, IA, USA.) using the Applied Biosystems 7500 Real Time PCR System (Applied Biosystems, Waltham, MA, USA). The following gene-specific PrimeTime^TM^ qPCR Probe Assays were used: *IL6* (assay ID: Hs.PT.58.40226675), *CXCL8* (assay ID: Hs.PT.58.39926886. g), and *ACTB* (assay ID: Hs.PT.39 a, 22214847).

### Statistical analysis

The results are expressed as the mean ± standard error of the mean obtained from at least three independent experimental replicates. One-way ANOVA was performed to evaluate differences across more than two experimental groups, followed by Tukey–Kramer’s honest significant difference (HSD) post hoc test. All statistical analyses were performed using the Statcel5 software (OMS, Tokyo, Japan). Statistical significance was set at p < 0.05.

### Segmented regression analysis

Segmented regression models were applied to the expression data of eight senescence-related genes (*CDKN1A, CDKN2A, IL6, CXCL8, MMP1, MMP3, SERPINE1*, and *LMNB1*) to identify transcriptional breakpoints along the PDL axis. For each gene, normalized expression values (log_2_(TPM + 1)) were plotted against PDL. Breakpoints were identified using a custom grid search approach, in which candidate PDL values were systematically tested as potential inflection points. For each candidate breakpoint, a two-piece linear model was fitted with a hinge at the breakpoint, and the breakpoint minimizing the residual sum of squares was selected. To assess the uncertainty of breakpoint estimates, bootstrap resampling (B = 1000) was performed, and 95% confidence intervals were calculated from the resulting distribution. All analyses were conducted in R (v4.5.2).

### cDNA library preparation and RNA-seq

Total RNA was extracted from seven independent samples using TRIzol Reagent (Thermo Fisher Scientific) according to the manufacturer’s protocol. RNA integrity was assessed on an Agilent 2200 TapeStation using an RNA ScreenTape (Agilent Technologies, Santa Clara, CA, USA). For library construction, an RIN value > 7 was selected from each cell sample at different senescence levels. Strand-specific sequencing libraries were prepared using the Illumina Stranded mRNA Prep Kit (Illumina, San Diego, CA, USA) according to the manufacturer’s protocol. The cDNA libraries were evaluated using a High-Sensitivity D1000 ScreenTape Kit on an Agilent 2200 TapeStation (Agilent Technologies). The prepared libraries were pooled and subjected to 150 bp paired-end sequencing on a DNBSEQ G-400 (BGI JAPAN, Hyogo, Japan).

### Bioinformatic analysis

Gene expression quantification and differential expression analysis were performed using two complementary approaches. An overview of these two workflows is illustrated in Figure 2B.

For the first approach, a GUI-based workflow developed in our laboratory, the Portable Pipeline, was employed in accordance with Senevirathna et al. [51]. Paired-end reads were filtered using PRINSEQ 0.20.4 [52], aligned, and mapped to the reference human genome (GCF_000001405.40_GRCh38.p14_genomic.fna) using HISAT2 2.1.0 [53]. The mapped sequences were assembled using StringTie 1.3.4 [54] with reference to the GFF of the same genome, considering splicing variants. Ballgown [55] was used to identify DEGs based on the output from StringTie and Cuffdiff [56] based on Cuffquant 2.2.1, respectively. DESeq2 was used to identify DEGs from the raw count matrix. Genes with an adjusted p-value < 0.05 were considered significantly differentially expressed. To enhance the existing genome annotation, functional annotation of the assembled transcripts was conducted using Trinotate [57]. These analyses were performed using Portable Pipeline-limux-v1.4.2 (https://github.com/c2997108/OpenPortablePipeline) by selecting “RNA-seq~HISAT2-StringTie-DEGanalysis” and running with default settings. Quality control of the raw sequencing data was performed using FastQC and MultiQC, and no problematic samples were identified. Sample-level variance was visually assessed using MDS plots generated by the Portable Pipeline. No apparent outliers were detected, and clustering patterns indicated high within-group consistency.

For the second approach, a general-purpose command-line workflow was applied. Raw reads were first filtered for adapter sequences and low-quality reads using atria version 4.0.0 [58]. Expression quantification followed Sullivan et al. [59], using kb-python version 0.29.5, kallisto version 0.51.1 [60, 61], and bustools version 0.45.1 [62]. The reference genome (GRCh38; Homo_sapiens.GRCh38.dna.primary_assembly.fa.gz) and corresponding GTF file (Homo_sapiens.GRCh38.113.gtf.gz) were obtained from Ensembl (https://www.ensembl.org/info/data/ftp/index.html). Sample-level expression matrices were integrated using Python version 3.13.1, pandas version 2.2.3, and numpy version 2.2.2. PCA was performed using matplotlib version 3.10.3 and scikit-learn version 1.7.0 for dimensionality reduction and sample-level visualization, which similarly confirmed consistent clustering and no outliers. DEGs were identified using PyDESeq2 version 0.5.1 [63] with an adjusted p-value threshold of < 0.05. Additionally, NMF (https://github.com/YiqunW/NMF/tree/master) was applied to extract metagenes and other features from the gene expression matrix. Violin plots were generated to visualize senescence-stage–dependent expression patterns of representative genes across three senescence stages (young, middle, and old). Statistical significance was assessed using one-way ANOVA followed by Tukey’s HSD test, with thresholds set at *p < 0.05, **p < 0.01, and ***p < 0.001. In addition to p-values, standardized effect sizes were calculated to quantify the magnitude of group differences. Cohen’s d was used to evaluate mean-based differences, whereas Cliff’s delta was applied as a non-parametric measure to assess distributional shifts. Detection rates (fraction of samples with TPM > 0) were also computed to complement effect size estimates. All statistical analyses were performed using R (v4.5.2). Additionally, Spearman’s correlation analysis was performed on log_2_(TPM + 1) values to assess sample-level similarity. Pairwise correlation coefficients were calculated across all samples, and the resulting matrix was visualized as a heatmap using the pheatmap package in R (v4.5.2). This analysis was used to confirm clustering patterns and to evaluate potential batch effects.

DEGs identified from each approach were subsequently analyzed for PPI networks using STRING version 12.0 [64] (https://string-db.org/) and for functional enrichment using Enrichr [65–67] (https://maayanlab.cloud/Enrichr/). PPI networks were clustered using k-means clustering, with the number of clusters determined empirically. Functional enrichment analysis focused on GO biological process terms and KEGG pathways to interpret the biological significance of the DEGs.

RNA-seq data have been deposited in the DDBJ Sequence Read Archive (DRA) under BioProject accession number PRJDB37596.

## Supplementary Material

Supplementary Figures

Supplementary Tables 1 and 2

Supplementary Table 3

Supplementary Table 4

Supplementary Table 5

Supplementary Table 6

Supplementary Table 7

Supplementary Table 8
