# Supplementary material for: Stage-dependent transcriptomic changes in human dermal fibroblast senescence model
Source: Aging (Albany NY). 2026 Apr 10;18(1):340–68. doi: 10.18632/aging.206371 (PMC13632856; doi:10.18632/aging.206371)
Supplement: Supplementary file 1 [file aging-18-1-206371-s001.pdf]

SUPPLEMENTARY FIGURES

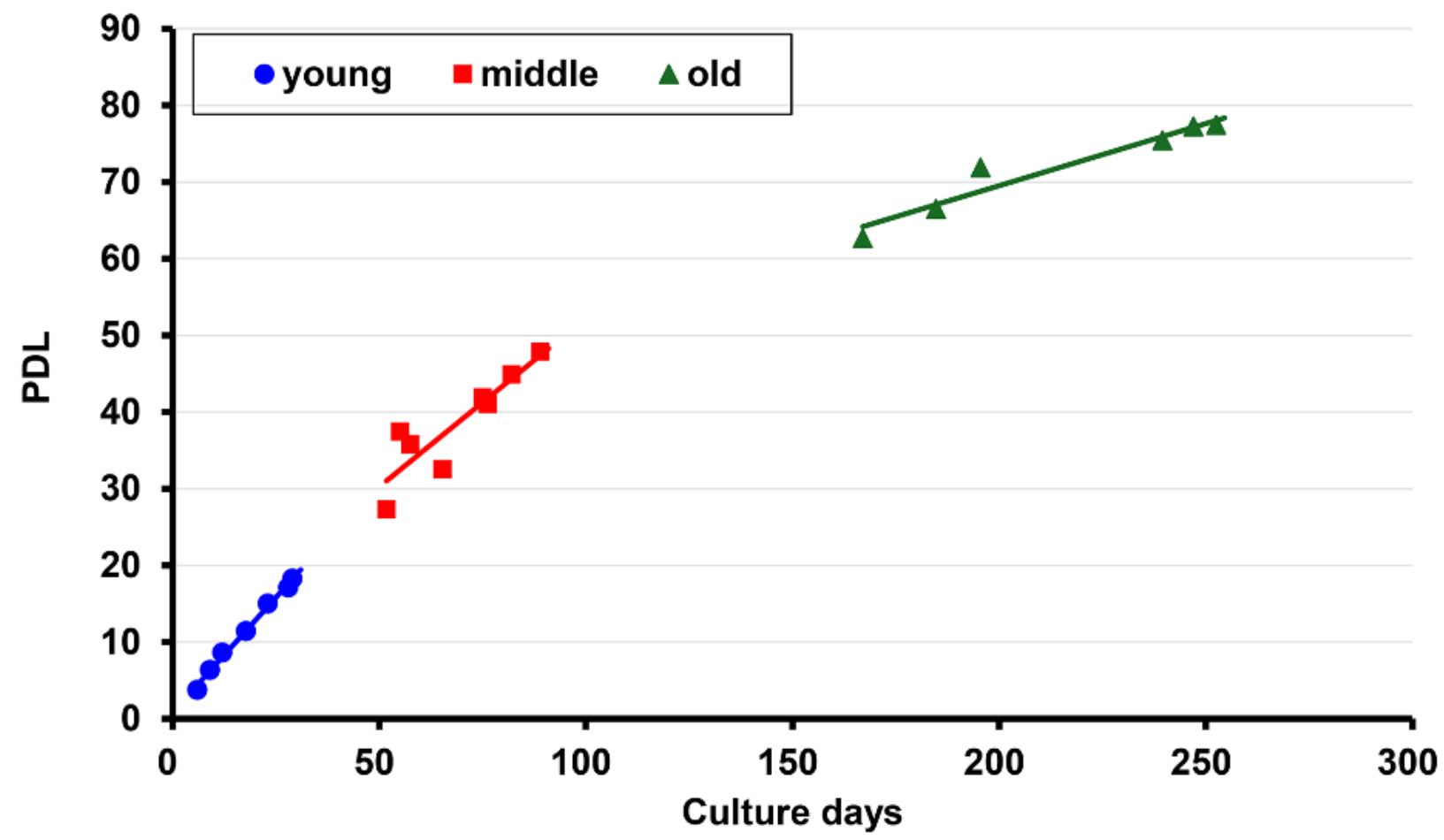

Supplementary Figure 1. Growth curve of normal human dermal fibroblasts (NHDFs) classified by senescence level. Growth curve showing cumulative population doubling level (PDL) across passages for NHDFs classified as young (PDL ≤ 20), middle (PDL 30–50), and old (PDL ≥ 60). The x-axis represents cumulative culture duration (days), whereas the y-axis shows cumulative PDL.

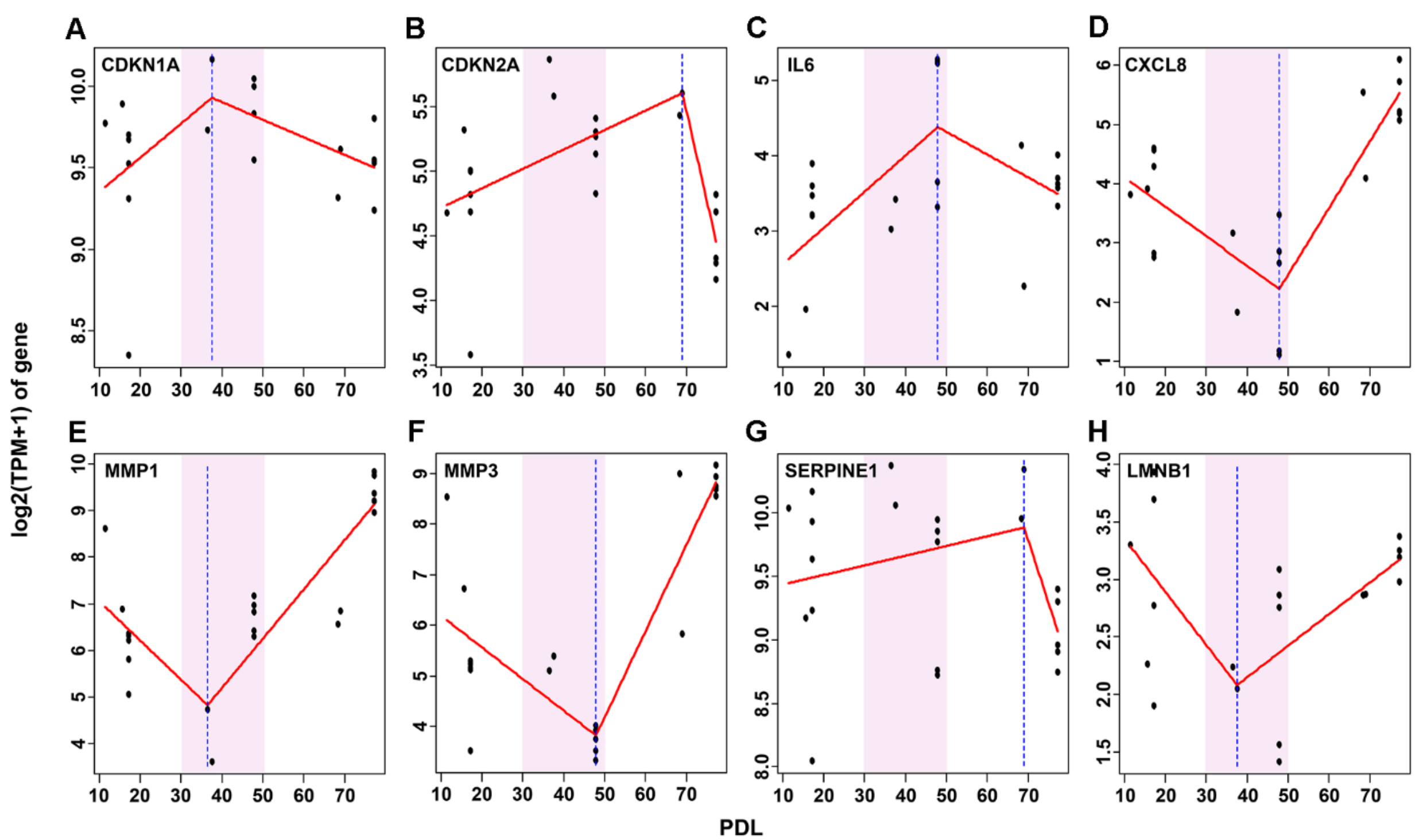

Supplementary Figure 2. Segmented regression analyses of senescence-related gene expression across PDLs. Expression levels of eight senescence-related genes—(**A**) *CDKN1A*, (**B**) *CDKN2A*, (**C**) *IL6*, (**D**) *CXCL8*, (**E**) *MMP1*, (**F**) *MMP3*, (**G**) *SERPINE1*, and (**H**) *LMNB1*—were analyzed using segmented regression models to identify transcriptional breakpoints along the PDL axis. Six genes (*CDKN1A, IL6, CXCL8, MMP1, MMP3,* and *LMNB1*) exhibited breakpoints within the middle group (PDL 30–50), supporting its biological relevance as a transitional phase of early senescence. In contrast, *CDKN2A* and *SERPINE1* showed breakpoints within the old group (PDL ≥ 60), specifically at PDL ≈ 67–69, consistent with late-stage senescence. The breakpoints near PDL 67–69 were interpreted as occurring within the lower range of the old group (PDL ≥ 60), consistent with late-stage senescence.

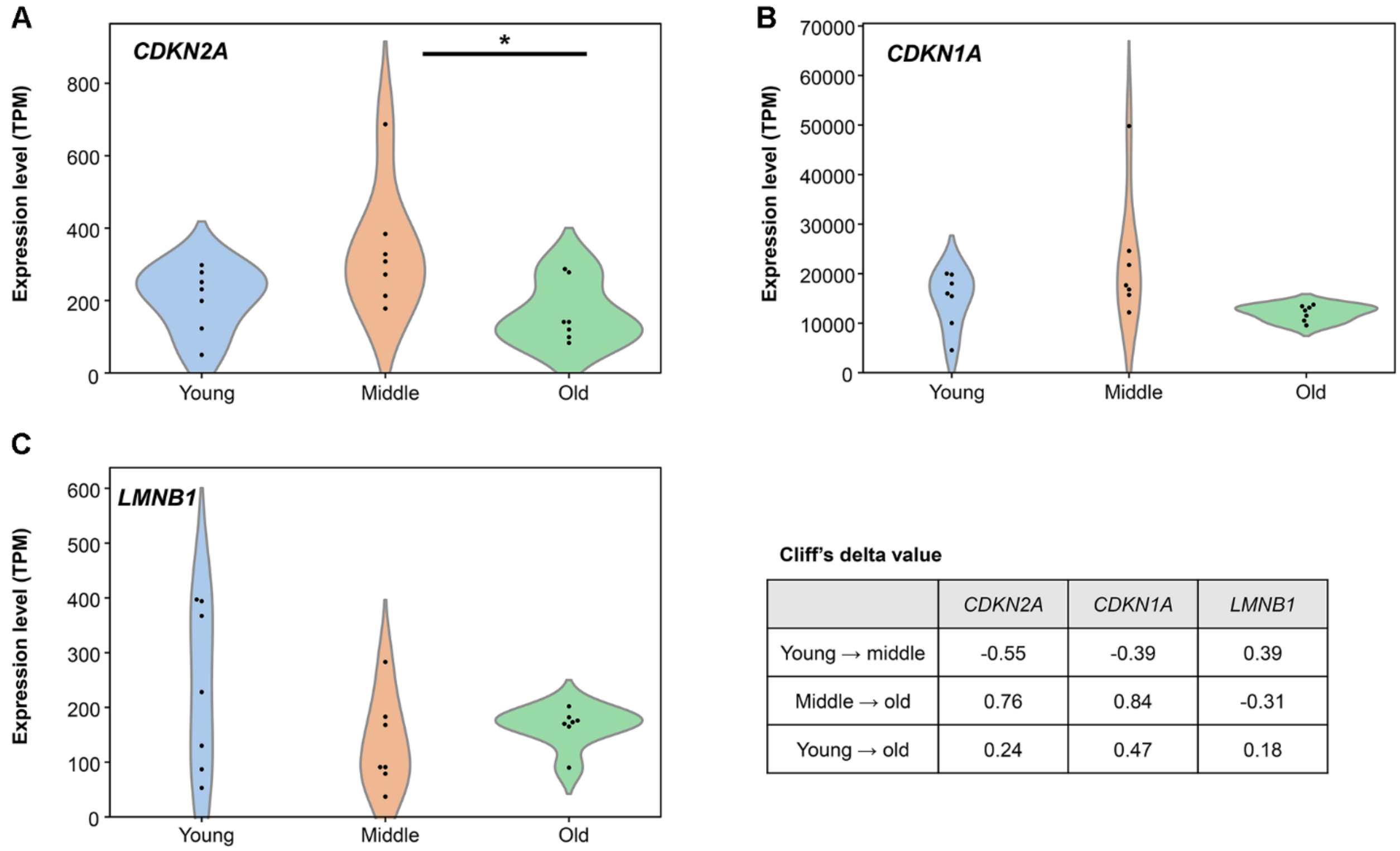

Cliff's delta value

	CDKN2A	*CDKN1A*	*LMNB1*
Young → middle	-0.55	-0.39	0.39
Middle → old	0.76	0.84	-0.31
Young → old	0.24	0.47	0.18

Supplementary Figure 3. Violin plots showing transcripts per million (TPM) distributions of canonical senescence markers (*CDKN2A*, *CDKN1A*, and *LMNB1*) across young, middle, and old groups. Violin plots for *CDKN2A* (**A**), *CDKN1A* (**B**), and *LMNB1* (**C**) are shown. Plots were generated using TPM values from the command-line workflow, which provides appropriate normalization for distributional visualization. Individual data points are overlaid. Statistical significance was assessed using one-way analysis of variance followed by Tukey's post-hoc test. A significant difference was observed only for *CDKN1A* between middle and old groups, whereas *CDKN2A* and *LMNB1* showed non-significant trends (*p < 0.05). Cliff's delta values for pairwise comparisons are shown in the inset table to illustrate effect sizes; positive values indicate higher expression in the first group, whereas negative values indicate higher expression in the second group.

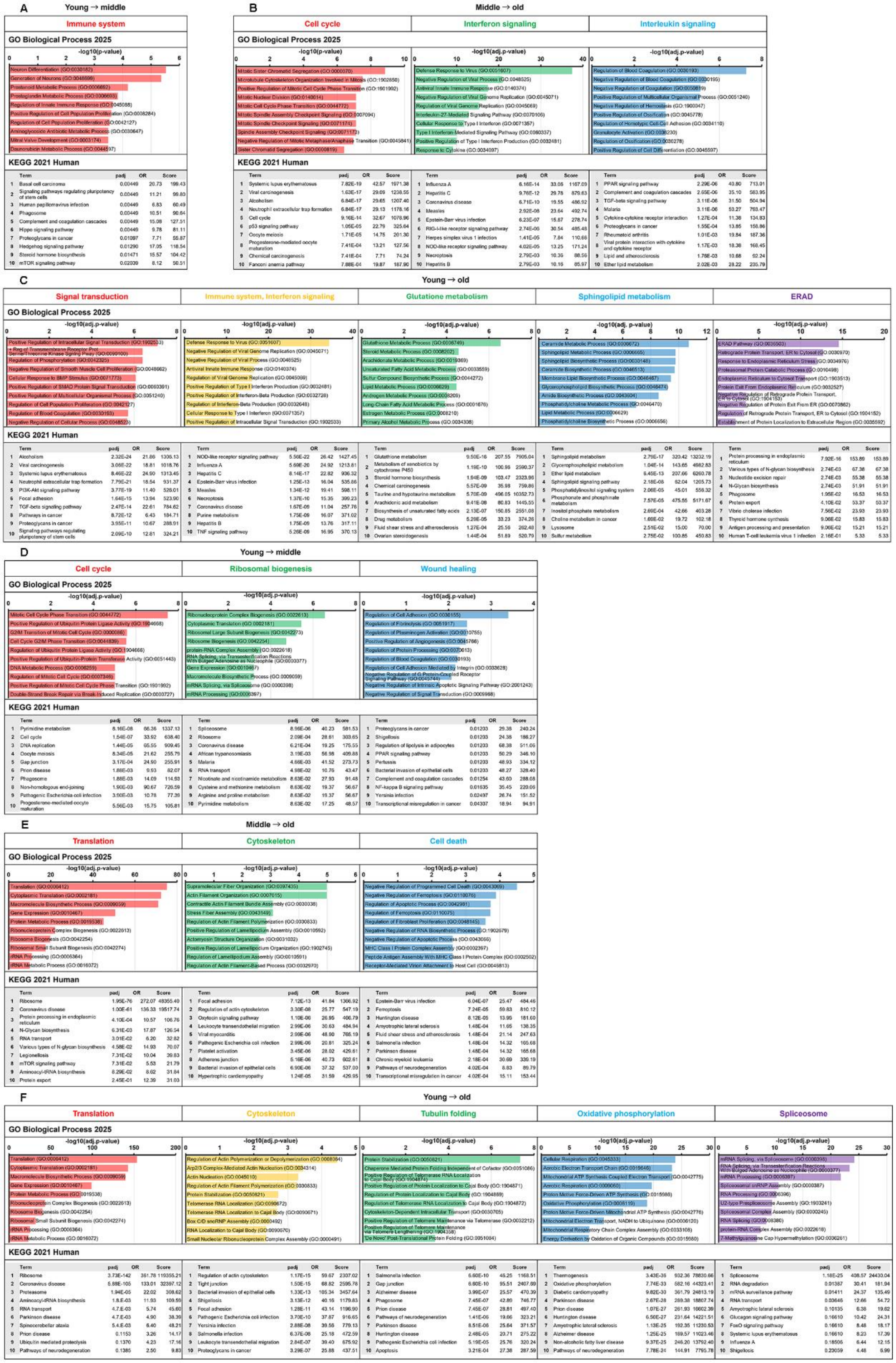

Supplementary Figure 4. Functional enrichment analysis of differentially expressed genes (DEGs). Functional enrichment analysis of DEGs identified from each pairwise comparison (from young to middle, middle to old, and young to old groups). The top 10 enriched pathways from Gene Ontology (GO) Biological Process (top) and Kyoto Encyclopedia of Genes and Genomes (KEGG) Human

(bottom) are shown. The lengths of the bars and score values represent the degree of enrichment. (**A**) Enrichment analysis of upregulated DEGs from young to middle. (**B**) Enrichment analysis of upregulated DEGs from middle to old. (**C**) Enrichment analysis of upregulated DEGs in the comparison of young to old. (**D**) Enrichment analysis of DEGs downregulated from young to middle. (**E**) Enrichment analysis of downregulated DEGs from middle to old. (**F**) Enrichment analysis of downregulated DEGs in the comparison of young to old.

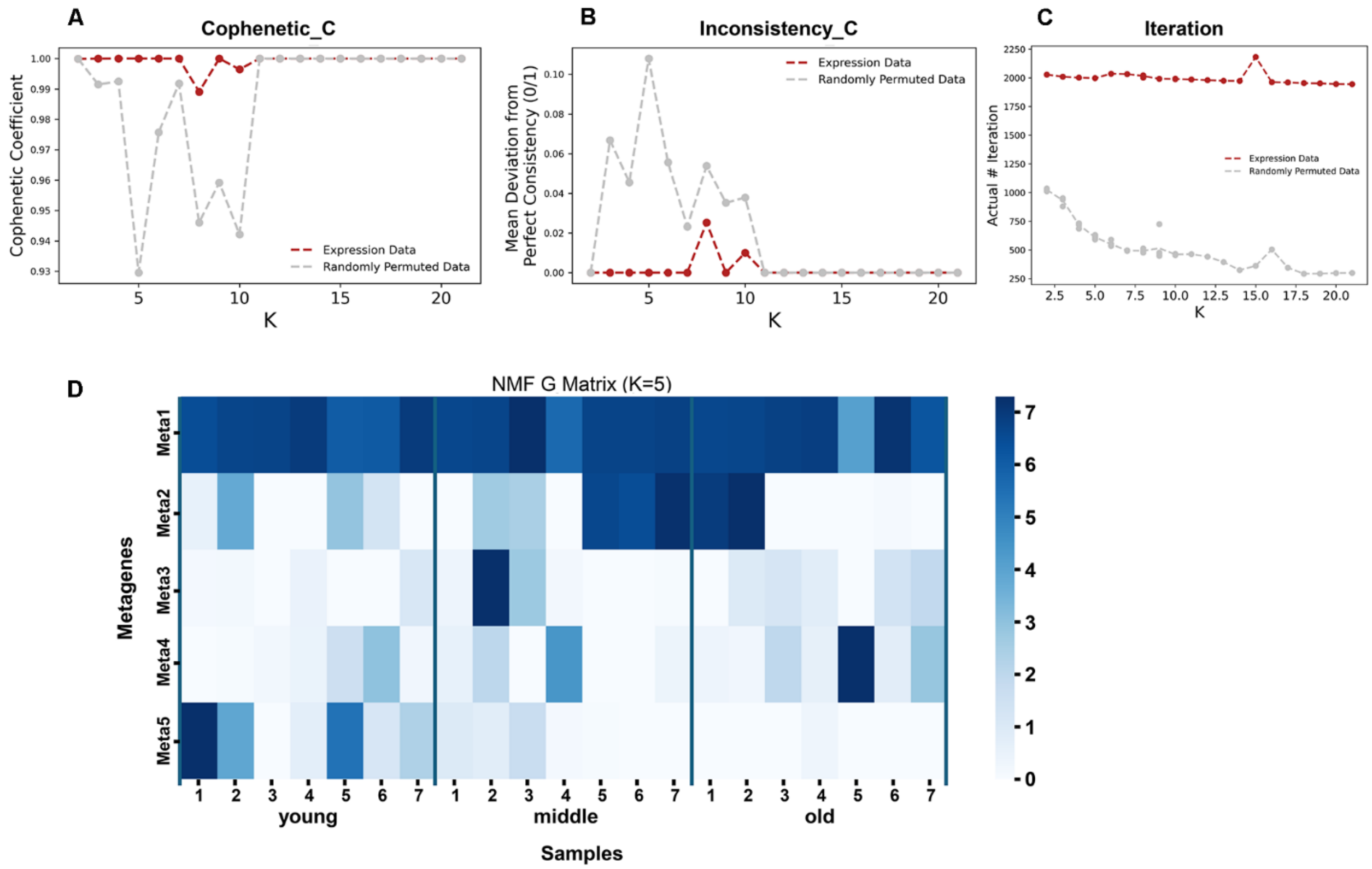

Supplementary Figure 5. Evaluation of clustering stability and consistency for non-negative matrix factorization (NMF) component selection. (**A**) Cophenetic coefficient across different values of K for expression data (red) and randomly permuted data (gray). The expression data maintained high cophenetic values across K, supporting a stable clustering structure. (**B**) Mean deviation from consensus clustering across K values. Expression data showed lower inconsistency compared to permuted data, indicating robust component extraction. (**C**) Actual number of iterations required for convergence across K values. Expression data exhibited stable iteration counts, whereas permuted data showed greater variability and reduced convergence efficiency. (**D**) Representative sample-by-metagene matrix at K = 5, showing insufficient separation of stage-dependent expression patterns. These metrics collectively support selecting K = 6 as the most stable and biologically interpretable solution for NMF decomposition.

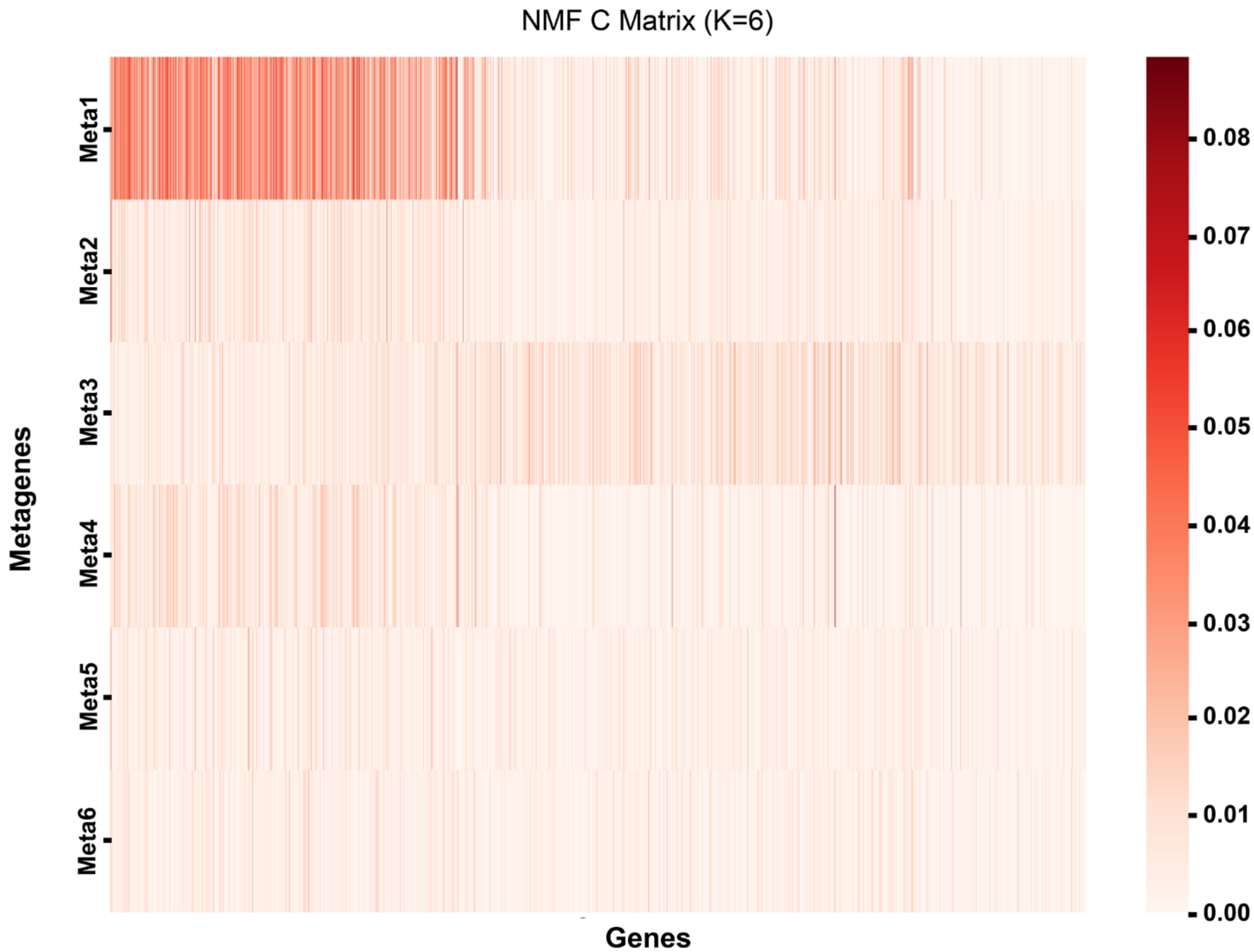

Supplementary Figure 6. NMF C matrix: Contribution of each gene to each metagene. The heat map represents the H matrix obtained from NMF, illustrating the relationship between metagenes and genes. The contribution of each gene to the six metagenes was identified through analysis (Meta1–Meta6). The intensity of the red color indicates the degree of contribution, with darker shades representing higher contributions. Rows correspond to metagenes, and columns represent individual genes.

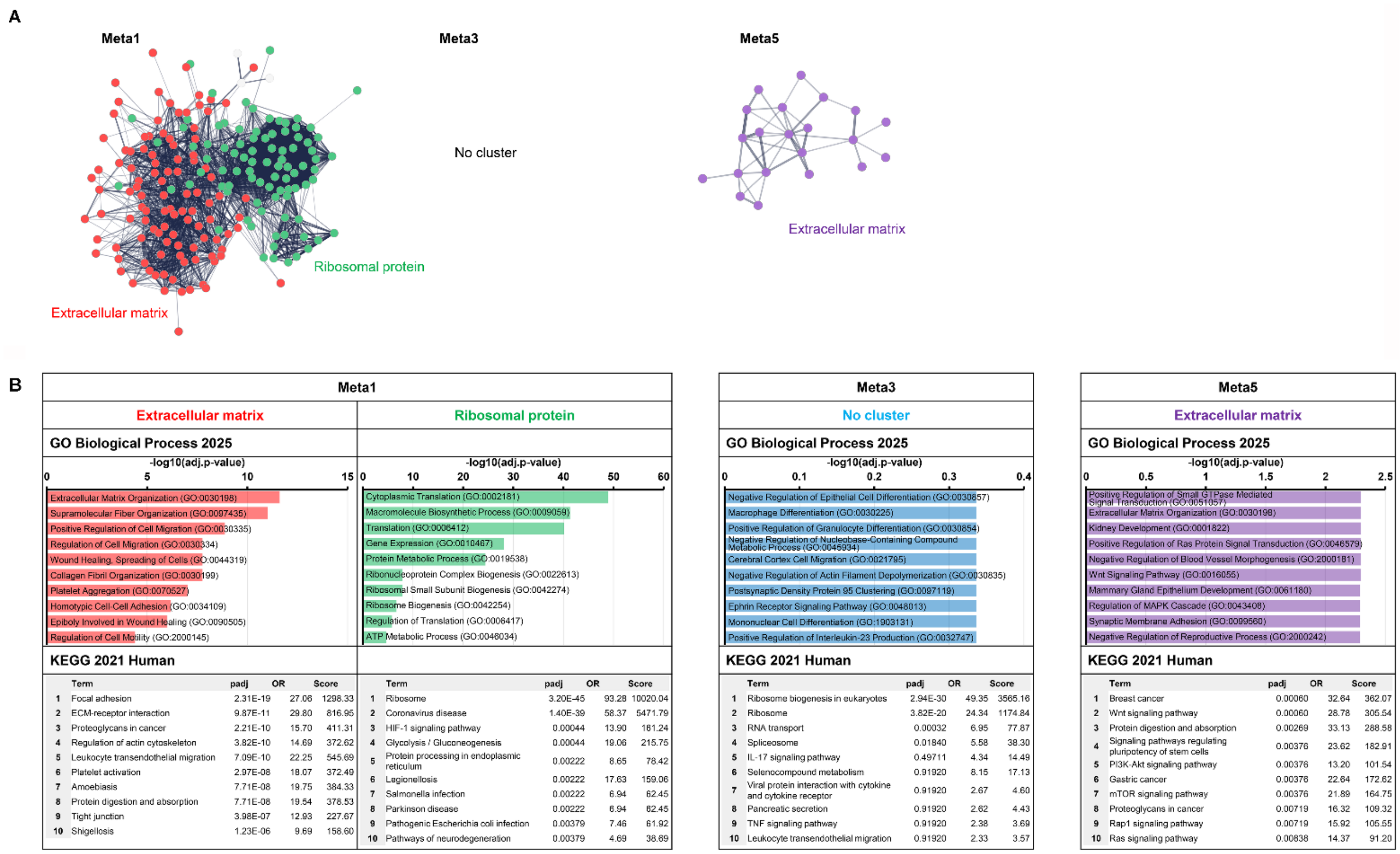

Supplementary Figure 7. Functional characterization of metagenes Meta1, Meta3, and Meta5. (**A**) STRING protein–protein interaction (PPI) networks of selected metagenes. The top genes from Meta1, Meta3, and Meta5 were used to construct PPI networks. Node clusters are highlighted in different colors and labeled with the corresponding UniProt keywords (Meta1: extracellular matrix/ribosomal protein; Meta3: no cluster; and Meta5: extracellular matrix). (**B**) Functional enrichment analysis of selected metagenes. Top 10 enriched pathways from GO Biological Process (top) and KEGG Human (bottom) for Meta1, Meta3, and Meta5, as determined by Enrichr. The length of the bars and score values represents the degree of enrichment.
