# Supplementary material for: Stage-dependent transcriptomic changes in human dermal fibroblast senescence model
Source: Aging (Albany NY). 2026 Apr 10;18(1):340–68. doi: 10.18632/aging.206371 (PMC13632856; doi:10.18632/aging.206371)
Supplement: Supplementary file 2 [file aging-18-1-206371-s002.pdf]

SUPPLEMENTARY TABLES

Please browse Full Text version to see the data of Supplementary Tables 3–5, 8.

Supplementary Table 1. Summary of culture duration and cumulative population doubling level (PDL) values used to construct growth curves.

Passage	Group	Cumulative culture days	Cumulative PDL
p0	young	6	3.76
p1	young	9	6.34
p2	young	12	8.60
p3	young	18	11.41
p4	young	23	15.00
p5	young	29	18.26
p6	young	28	17.13
p7	middle	52	27.31
p8	middle	65	32.53
p9	middle	55	37.44
p10	middle	58	35.79
p11	middle	76	41.02
p12	middle	75	41.89
p13	middle	82	44.89
p14	middle	89	47.89
p12	old	167	62.65
p13	old	185	66.48
p14	old	196	71.88
p15	old	240	75.45
p16	old	253	77.45
p17	old	247	77.24

Supplementary Table 1 shows the correspondence between passage number, cumulative PDL, culture duration, and classification into young, middle, and old groups. Classification was based on cumulative PDL to more accurately represent biological aging, as passage number does not always reflect the actual replication history (e.g., repeated subculture at low seeding density). This table supports Supplementary Figure 1.

Supplementary Table 2. Estimated breakpoints and bootstrap confidence intervals for senescence-related genes.

Gene	Breakpoint		Bootstrap 95% CI		Group
	segmented model	grid search, visualized	lower	upper	
CDKN1A	39.7	37.5	15.6	54.7	middle
CDKN2A	67.4	69.0	28.2	69	old
IL6	19.6	47.9	17.3	53.2	middle
CXCL8	45.3	47.9	28.7	61.8	middle
MMP1	22.4	36.5	19.7	68.4	middle
MMP3	47.9	47.9	19.5	62.6	middle
SERPINE1	69.0	69.0	15.6	69	old
LMNB1	39.1	37.5	18	59.9	middle

Breakpoints were estimated using segmented regression models fitted to $\log_2(TPM + 1)$ expression values across cumulative PDLs. Grid search breakpoints correspond to the values visualized in Supplementary Figure 2 (blue dashed lines), selected by minimizing residual sum of squares. Bootstrap confidence intervals (B = 1000) reflect uncertainty in breakpoint estimation.
