# Supplementary material for: Stage-dependent transcriptomic changes in human dermal fibroblast senescence model
Source: Aging (Albany NY). 2026 Apr 10;18(1):340–68. doi: 10.18632/aging.206371 (PMC13632856; doi:10.18632/aging.206371)
Supplement: Supplementary file 3 [file aging-18-1-206371-s006.pdf]

Supplementary Table 6. Expression summary of canonical senescence markers (*CDKN2A*, *CDKN1A*, and *LMNB1*) across two analytical pipelines.

Gene	Alias	Pipeline	Comparison	Metric	Group 1	Group 2	log2FC	padj
CDKN2A	p16	Portable	Y vs. M	avg RPKM	NA	NA	NA	NA
CDKN2A	p16	Portable	M vs. O	avg RPKM	NA	NA	NA	NA
CDKN2A	p16	Portable	Y vs. O	avg RPKM	NA	NA	NA	NA
CDKN2A	p16	Command-line	Y vs. M	baseMean	—	—	NA	NA
CDKN2A	p16	Command-line	M vs. O	baseMean	—	—	-1.28	1.10E-10
CDKN2A	p16	Command-line	Y vs. O	baseMean	—	—	-1.01	6.82E-07
CDKN1A	p21	Portable	Y vs. M	avg RPKM	NA	NA	NA	NA
CDKN1A	p21	Portable	M vs. O	avg RPKM	346.60	263.24	-0.40	2.47E-03
CDKN1A	p21	Portable	Y vs. O	avg RPKM	NA	NA	NA	NA
CDKN1A	p21	Command-line	Y vs. M	baseMean	—	—	NA	NA
CDKN1A	p21	Command-line	M vs. O	baseMean	—	—	-0.83	7.13E-06
CDKN1A	p21	Command-line	Y vs. O	baseMean	—	—	-0.61	1.27E-03
LMNB1	Lamin B1	Portable	Y vs. M	avg RPKM	NA	NA	NA	NA
LMNB1	Lamin B1	Portable	M vs. O	avg RPKM	NA	NA	NA	NA
LMNB1	Lamin B1	Portable	Y vs. O	avg RPKM	NA	NA	NA	NA
LMNB1	Lamin B1	Command-line	Y vs. M	baseMean	—	—	-1.28	3.46E-05
LMNB1	Lamin B1	Command-line	M vs. O	baseMean	—	—	NA	NA
LMNB1	Lamin B1	Command-line	Y vs. O	baseMean	—	—	-0.79	8.41E-03

Differential expression summary for *CDKN2A*, *CDKN1A*, and *LMNB1* were extracted from the DEG lists in Supplementary Table 3 (Portable Pipeline) and Supplementary Table 4 (Command-line), covering all pairwise comparisons (Y vs. M, M vs. O, Y vs. O).
Comparison represents group contrasts: young−middle, middle−old, and young−old.
Metric differs by pipeline: avg RPKM (Portable Pipeline) and baseMean (Command-line).
Group 1/Group 2 values are shown only for Portable comparisons with padj < 0.05; for Command-line results, these fields are denoted with "—".
NA indicates non-significant results (padj ≥ 0.05).
