# Supplementary material for: Stage-dependent transcriptomic changes in human dermal fibroblast senescence model
Source: Aging (Albany NY). 2026 Apr 10;18(1):340–68. doi: 10.18632/aging.206371 (PMC13632856; doi:10.18632/aging.206371)
Supplement: Supplementary file 4 [file aging-18-1-206371-s008.pdf]

Supplementary Table 8. Statistical validation of representative genes in Figure 7.

Gene	ANOVA p	Comparison (Tukey)	Mean diff	95% CI		p-adj	Sig.	Cohen's d	Effect size label	DetectRate (Y/M/O)
				lower	upper					
IFIT1	0.00025	young → middle	+107.29	-1340.42	1554.99	0.9	–	1.048	large	1 / 1 / 1
		middle → old	+2509.86	1062.15	3957.56	0.001	✓	1.931	large	
		young → old	+2617.14	1169.44	4064.85	0.001	✓	2.019	large	
MX1	0.00032	young → middle	+386.29	-4045.94	4818.51	0.9	–	0.925	large	1 / 1 / 1
		middle → old	+7465.43	3033.21	11897.65	0.0012	✓	1.876	large	
		young → old	+7851.71	3419.49	12283.94	0.001	✓	1.983	large	
OAS1	0.00023	young → middle	+22.86	-977.66	1023.37	0.9	–	0.77	medium	0.43 / 1 / 1
		middle → old	+1775.43	774.91	2775.95	0.001	✓	1.976	large	
		young → old	+1798.29	797.77	2798.80	0.001	✓	2.002	large	
EIF3CL	0.33571	young → middle	-46.00	-425.18	333.18	0.9	–	-0.14	negligible	1 / 1 / 1
		middle → old	-169.00	-548.18	210.18	0.5055	–	-0.56	medium	
		young → old	-215.00	-594.18	164.18	0.3396	–	-1.195	large	
EEF1D	0.34302	young → middle	+219.57	-3765.34	4204.49	0.9	–	0.062	negligible	1 / 1 / 1
		middle → old	-2140.00	-6124.92	1844.92	0.3778	–	-0.628	medium	
		young → old	-1920.43	-5905.34	2064.49	0.4534	–	-1.581	large	
RPLP2	0.20102	young → middle	+174.71	-7085.63	7435.06	0.9	–	0.027	negligible	1 / 1 / 1
		middle → old	-4703.86	-11964.20	2556.49	0.2501	–	-0.776	medium	
		young → old	-4529.14	-11789.49	2731.20	0.2748	–	-1.727	large	
MMP9	0.06970	young → middle	+10.43	-4.84	25.69	0.2171	–	0.762	medium	0.86 / 0.86 / 0.57
		middle → old	-14.43	-29.69	0.84	0.0658	–	-1.08	large	
		young → old	-4.00	-19.27	11.27	0.771	–	-1.265	large	
SULF1	0.30319	young → middle	+1529.43	-959.71	4018.57	0.2849	–	0.748	medium	1 / 1 / 1
		middle → old	-504.29	-2993.42	1984.85	0.857	–	-0.226	small	
		young → old	+1025.14	-1464.00	3514.28	0.5544	–	1.133	large	
PCDHGB2	0.03584	young → middle	+33.71	-1.21	68.63	0.0595	–	1.088	large	0.86 / 1 / 1
		middle → old	-33.57	-68.49	1.35	0.0607	–	-1.163	large	
		young → old	+0.14	-34.78	35.06	0.9	–	0.011	negligible	

Mean differences are reported according to the arrow notation used in the main text (young→middle = middle−young; middle→old = old−middle; young→old = old−young). Tukey honest significant difference pairwise comparisons, adjusted p-values (padj), Cohen's d effect sizes, and detect rates are provided.
