# Supplementary material for: Stage-dependent transcriptomic changes in human dermal fibroblast senescence model
Source: Aging (Albany NY). 2026 Apr 10;18(1):340–68. doi: 10.18632/aging.206371 (PMC13632856; doi:10.18632/aging.206371)
Supplement: Supplementary file 5 [file aging-18-1-206371-s007.docx]

Supplementary Table 7. Top five enriched pathways in each functional cluster identified by STRING analysis.

| **Comparison** | **Expression** | **Cluster** | **#Term ID** | **Term description** | **Gene count** | **FDR** |
| --- | --- | --- | --- | --- | --- | --- |
| Young → middle | Upregulate | Cluster 1 | HSA-168256 | Immune system | 21 | 0.0059 |
|  |  |  | HSA-1474244 | Extracellular matrix organization | 7 | 0.0399 |
|  |  |  | HSA-168249 | Innate immune system | 13 | 0.0399 |
|  |  |  | HSA-381426 | Regulation of insulin-like growth factor (IGF) transport and uptake by Insulin-like growth factor binding proteins (IGFBPs) | 5 | 0.0399 |
|  |  |  | HSA-8957275 | Post-translational protein phosphorylation | 5 | 0.0399 |
|  | Downregulate | Cluster 1 | HSA-69278 | Cell cycle, mitotic | 22 | 2.63E-20 |
|  |  |  | HSA-68886 | M phase | 13 | 7.78E-10 |
|  |  |  | HSA-68882 | Mitotic anaphase | 11 | 1.37E-09 |
|  |  |  | HSA-2467813 | Separation of sister chromatids | 10 | 3.65E-09 |
|  |  |  | HSA-2500257 | Resolution of sister chromatid cohesion | 8 | 9.68E-08 |
|  |  | Cluster 2 | HSA-8953854 | Metabolism of RNA | 12 | 5.15E-09 |
|  |  |  | HSA-6791226 | Major pathway of rRNA processing in the nucleolus and cytosol | 6 | 0.0000504 |
|  |  |  | HSA-156827 | L13a-mediated translational silencing of ceruloplasmin expression | 5 | 0.0000686 |
|  |  |  | HSA-72706 | GTP hydrolysis and joining of the 60S ribosomal subunit | 5 | 0.0000686 |
|  |  |  | HSA-9754678 | SARS-CoV-2 modulates host translation machinery | 4 | 0.0000832 |
|  |  | Cluster 3 | WP5055 | Burn wound healing | 3 | 0.0298 |
| Middle → old | Upregulate | Cluster 1 | HSA-1640170 | Cell cycle | 29 | 2.35E-18 |
|  |  |  | HSA-69278 | Cell cycle, mitotic | 27 | 2.35E-18 |
|  |  |  | HSA-69620 | Cell cycle checkpoints | 18 | 1.75E-13 |
|  |  |  | HSA-68886 | M phase | 16 | 5.59E-09 |
|  |  |  | HSA-2500257 | Resolution of sister chromatid cohesion | 11 | 5.95E-09 |
|  |  | Cluster 2 | HSA-913531 | Interferon signaling | 28 | 7.91E-34 |
|  |  |  | HSA-909733 | Interferon alpha/beta signaling | 19 | 9.83E-27 |
|  |  |  | HSA-1280215 | Cytokine signaling in immune system | 33 | 1.29E-26 |
|  |  |  | HSA-168256 | Immune system | 40 | 2.39E-20 |
|  |  |  | HSA-1169410 | Antiviral mechanism by IFN-stimulated genes | 14 | 5.49E-17 |
|  |  | Cluster 3 | HSA-1474244 | Extracellular matrix organization | 11 | 4.84E-07 |
|  |  |  | HSA-6785807 | Interleukin-4 and interleukin-13 signaling | 7 | 0.0000142 |
|  |  |  | HSA-75205 | Dissolution of fibrin clot | 4 | 0.0000592 |
|  |  |  | HSA-449147 | Signaling by interleukins | 10 | 0.0000942 |
|  |  |  | HSA-109582 | Hemostasis | 11 | 0.00012 |
|  | Downregulate | Cluster 1 | HSA-156842 | Eukaryotic translation elongation | 46 | 5.58E-80 |
|  |  |  | HSA-72766 | Translation | 55 | 4.17E-79 |
|  |  |  | HSA-156902 | Peptide chain elongation | 44 | 1.86E-76 |
|  |  |  | HSA-156827 | L13a-mediated translational silencing of ceruloplasmin expression | 44 | 4.36E-73 |
|  |  |  | HSA-1799339 | SRP-dependent cotranslational protein targeting to membrane | 44 | 4.36E-73 |
|  |  | Cluster 2 | HSA-194315 | Signaling by Rho GTPases | 11 | 0.00029 |
|  |  |  | HSA-446353 | Cell-extracellular matrix interactions | 4 | 0.00029 |
|  |  |  | HSA-9012999 | RHO GTPase cycle | 9 | 0.00029 |
|  |  |  | HSA-9664422 | FCGR3A-mediated phagocytosis | 5 | 0.00029 |
|  |  |  | HSA-2029480 | Fcgamma receptor (FCGR) dependent phagocytosis | 5 | 0.00046 |
|  |  | Cluster 3 | HSA-8852276 | The role of GTSE1 in G2/M progression after G2 checkpoint | 7 | 7.44E-07 |
|  |  |  | HSA-69275 | G2/M transition | 8 | 7.22E-06 |
|  |  |  | HSA-168256 | Immune system | 18 | 8.32E-06 |
|  |  |  | HSA-2262752 | Cellular responses to stress | 12 | 9.07E-06 |
|  |  |  | HSA-449147 | Signaling by interleukins | 9 | 0.0000875 |
| Young → old | Upregulate | Cluster 1 | HSA-162582 | Signal transduction | 73 | 1.66E-14 |
|  |  |  | HSA-1474244 | Extracellular matrix organization | 26 | 1.98E-13 |
|  |  |  | HSA-109582 | Hemostasis | 30 | 6.27E-10 |
|  |  |  | HSA-9006931 | Signaling by nuclear receptors | 21 | 6.27E-10 |
|  |  |  | HSA-2559583 | Cellular senescence | 17 | 1.11E-09 |
|  |  | Cluster 2 | HSA-1430728 | Metabolism | 24 | 2.32E-17 |
|  |  |  | HSA-211859 | Biological oxidations | 10 | 1.79E-10 |
|  |  |  | HSA-556833 | Metabolism of lipids | 14 | 1.79E-10 |
|  |  |  | HSA-8978868 | Fatty acid metabolism | 9 | 1.07E-09 |
|  |  |  | HSA-156590 | Glutathione conjugation | 6 | 6.15E-09 |
|  |  | Cluster 3 | HSA-556833 | Metabolism of lipids | 18 | 1.72E-18 |
|  |  |  | HSA-1430728 | Metabolism | 21 | 9.08E-16 |
|  |  |  | HSA-428157 | Sphingolipid metabolism | 9 | 7.44E-13 |
|  |  |  | HSA-1660661 | Sphingolipid de novo biosynthesis | 7 | 6.83E-11 |
|  |  |  | HSA-1483206 | Glycerophospholipid biosynthesis | 8 | 7.1E-10 |
|  |  | Cluster 4 | HSA-556833 | Metabolism of lipids | 18 | 1.72E-18 |
|  |  |  | HSA-1430728 | Metabolism | 21 | 9.08E-16 |
|  |  |  | HSA-428157 | Sphingolipid metabolism | 9 | 7.44E-13 |
|  |  |  | HSA-1660661 | Sphingolipid de novo biosynthesis | 7 | 6.83E-11 |
|  |  |  | HSA-1483206 | Glycerophospholipid biosynthesis | 8 | 7.1E-10 |
|  |  | Cluster 5 | HSA-532668 | N-glycan trimming in the ER and calnexin/calreticulin cycle | 7 | 8.39E-12 |
|  |  |  | HSA-446203 | Asparagine N-linked glycosylation | 9 | 2.8E-09 |
|  |  |  | HSA-901042 | Calnexin/calreticulin cycle | 4 | 9.99E-06 |
|  |  |  | HSA-597592 | Post-translational protein modification | 10 | 0.0000515 |
|  |  |  | HSA-392499 | Metabolism of proteins | 11 | 0.0000576 |
|  | Downregulate | Cluster 1 | HSA-72766 | Translation | 101 | 1.59E-140 |
|  |  |  | HSA-156842 | Eukaryotic translation elongation | 77 | 4.28E-125 |
|  |  |  | HSA-156902 | Peptide chain elongation | 74 | 6.49E-120 |
|  |  |  | HSA-72689 | Formation of a pool of free 40S subunits | 75 | 3.86E-119 |
|  |  |  | HSA-156827 | L13a-mediated translational silencing of ceruloplasmin expression | 76 | 5.29E-119 |
|  |  | Cluster 2 | HSA-195258 | RHO GTPase effectors | 10 | 1.31E-07 |
|  |  |  | HSA-422475 | Axon guidance | 12 | 1.31E-07 |
|  |  |  | HSA-5663213 | RHO GTPases activate WASPs and WAVEs | 6 | 1.31E-07 |
|  |  |  | HSA-2682334 | EPH-ephrin signaling | 7 | 1.42E-07 |
|  |  |  | HSA-446353 | Cell-extracellular matrix interactions | 5 | 1.42E-07 |
|  |  | Cluster 3 | HSA-389957 | Prefoldin mediated transfer of substrate to CCT/TriC | 7 | 1.57E-11 |
|  |  |  | HSA-389960 | Formation of tubulin folding intermediates by CCT/TriC | 7 | 1.57E-11 |
|  |  |  | HSA-3371497 | HSP90 chaperone cycle for steroid hormone receptors (SHR) in the presence of ligand | 6 | 4.38E-08 |
|  |  |  | HSA-5617833 | Cilium assembly | 8 | 4.38E-08 |
|  |  |  | HSA-1643685 | Disease | 14 | 6.42E-07 |
|  |  | Cluster 4 | HSA-1428517 | The citric acid (TCA) cycle and respiratory electron transport | 18 | 5.91E-30 |
|  |  |  | HSA-163200 | Respiratory electron transport, ATP synthesis by chemiosmotic coupling, and heat production by uncoupling proteins | 17 | 5.91E-30 |
|  |  |  | HSA-611105 | Respiratory electron transport | 14 | 7E-24 |
|  |  |  | HSA-6799198 | Complex I biogenesis | 9 | 8.18E-15 |
|  |  |  | HSA-1430728 | Metabolism | 19 | 1.73E-13 |
|  |  | Cluster 5 | HSA-72203 | Processing of capped intron-containing pre-mRNA | 17 | 2.69E-26 |
|  |  |  | HSA-72163 | mRNA splicing - major pathway | 15 | 1.18E-23 |
|  |  |  | HSA-8953854 | Metabolism of RNA | 18 | 1.22E-22 |
|  |  |  | HSA-73856 | RNA polymerase II transcription termination | 7 | 1.58E-10 |
|  |  |  | HSA-72187 | mRNA 3-end processing | 5 | 1.11E-06 |

For each comparison (young → middle, middle → old, and young → old), differentially expressed genes (DEGs) were analyzed using STRING to identify protein–protein interaction (PPI) networks. Functional clusters within the networks were annotated using Reactome pathways. The top five enriched pathways for each cluster are listed with their respective gene counts and false discovery rate (FDR) values.
